# Small extracellular vesicles from malignant ascites of patients with advanced ovarian cancer provide insights into the dynamics of the extracellular matrix

**DOI:** 10.1002/1878-0261.13110

**Published:** 2021-10-27

**Authors:** Barbara Bortot, Maura Apollonio, Enrico Rampazzo, Francesco Valle, Marco Brucale, Andrea Ridolfi, Blendi Ura, Riccardo Addobbati, Giovanni Di Lorenzo, Federico Romano, Francesca Buonomo, Chiara Ripepi, Giuseppe Ricci, Stefania Biffi

**Affiliations:** ^1^ Department of Medical Genetics Institute for Maternal and Child Health IRCCS Burlo Garofolo Trieste Italy; ^2^ Pediatric Department Institute for Maternal and Child Health IRCCS Burlo Garofolo Trieste Italy; ^3^ Department of Chemistry "Giacomo Ciamician" University of Bologna Italy; ^4^ Consorzio Sistemi a Grande Interfase Department of Chemistry University of Firenze Italy; ^5^ Consiglio Nazionale delle Ricerche Istituto per lo Studio dei Materiali Nanostrutturati (CNRISMN) Bologna Italy; ^6^ Department of Chemistry University of Firenze Italy; ^7^ Obstetrics and Gynecology Institute for Maternal and Child Health IRCCS Burlo Garofolo Trieste Italy; ^8^ Department of Clinical Toxicology Institute for Maternal and Child Health IRCCS Burlo Garofolo Trieste Italy; ^9^ Clinical Department of Medical Surgical and Health Sciences University of Trieste Italy

**Keywords:** ascites, chemotherapy, extracellular matrix, extracellular vesicles, fibronectin, ovarian cancer

## Abstract

The exact role of malignant ascites in the development of intraperitoneal metastases remains unclear, and the mechanisms by which extracellular vesicles (EVs) promote tumor progression in the pre‐metastatic niche have not been fully discovered. In this study, we characterized ascites from high‐grade epithelial ovarian cancer patients. Small‐EVs (30–150 nm) were isolated from two sources—the bulk ascites and the ascitic fluid‐derived tumor cell cultures—and assessed with a combination of imaging, proteomic profiling, and protein expression analyses. In addition, Gene Ontology and pathway analysis were performed using different databases and bioinformatic tools. The results proved that the small‐EVs derived from the two sources exhibited significantly different stiffness and size distributions. The bulk ascitic fluid‐derived small‐EVs were predominantly involved in the complement and coagulation cascade. Small‐EVs derived from ascites cell cultures contained a robust proteomic profile of extracellular matrix remodeling regulators, and we observed an increase in transforming growth factor‐β‐I (TGFβI), plasminogen activator inhibitor 1 (PAI‐1), and fibronectin expression after neoadjuvant chemotherapy. When measured in the two sources, we demonstrated that fibronectin exhibited opposite expression patterns in small‐EVs in response to chemotherapy. These findings highlight the importance of an ascites cell isolation workflow in investigating the treatment‐induced cancer adaption processes.

## Graphical abstract

The mechanisms by which extracellular vesicles (EVs) promote ovarian cancer progression in the pre‐metastatic niche have not been fully discovered. This study showed that sampling ascites before and after chemotherapy allows characterizing small‐EVs released by tumor cells, reflecting their biological status. Small‐EVs derived from ascitic fluid‐derived tumor cell cultures contained a robust proteomic profile of extracellular matrix remodeling regulators.

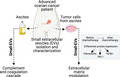

**Abbreviations**
b‐FGF: fibroblast growth factor basic
BSA: bovine serum albumin
CA: contact angle
CEUR: regional ethical committee of Friuli‐Venezia Giulia
CRS: chemotherapy response score
DLS: dynamic light scattering
ECM: extracellular matrix
EGF: epidermal growth factor
EOC: epithelial ovarian cancer cell
EVs: extracellular vesicles
FIGO: International Federation of Gynecology and Obstetrics
GO: Gene Ontology
GO‐BP: Gene Ontology ‐Biological Process
GO‐CC: Gene Ontology‐Cellular Component
GO‐MF: Gene Ontology‐Molecular Function
H&E: hematoxylin and eosin
HESFM: human endothelial serum‐free medium
HMW HA: high‐molecular‐weight hyaluronic acid
HRP: horseradish peroxidase
IDS: interval debulking surgery
IGF: insulin‐like growth factor
KEGG: Kyoto Encyclopedia of Genes and Genomes
MCK: multicytokeratin
MUC16: Mucin‐16
OC: ovarian cancer
PdI: Polydispersion Index
PDS: primary debulking surgery
PIV: total predictive index value
PLR: platelet‐to‐lymphocyte ratio
PNR: platelet‐to‐neutrophil ratio
REAC: Reactome
SD: standard deviation
TEM: transmission electronic microscopy
TME: tumor microenvironment

## 1 Introduction

High‐grade epithelial ovarian cancer is one of the most lethal gynecological malignancies, with a 5‐year survival rate of 40–45% [1]. The poor prognosis in over 70% of patients with ovarian cancer (OC) is mainly due to a diagnosis in advanced stages of the disease (FIGO stage III‐IV) when the extensive intraperitoneal dissemination has already occurred [2, 3]. The most important prognostic factor is the response to platinum‐based chemotherapy, as chemotherapy resistance is a significant challenge in OC management [4]. OC patients who recur within six months of ending platinum‐based chemotherapy are considered ‘platinum‐resistant’ and have poor prognoses [5].

During the peritoneal dissemination process, OC cells dissociate from the primary site of origin, which may be the ovary or fallopian tube [6]. After exfoliation from the primary tumor and survival, the surviving cancer cells spread within the peritoneal cavity by peritoneal fluid flow and colonize the surface of the peritoneal organs [7]. Notably, the *omentum* is the predominant homing site of metastasizing tumor cells and supports their growth by immunological and metabolic mechanisms [8].

The metastatic spread occurs gradually, at first with the formation of a pre‐metastatic niche arising before the macroscopic cancer cell invasion [9]. The pre‐metastatic niche is a distant tumor microenvironment (TME) primed by different factors, including exosomes released by the primary tumor [10, 11, 12]. Exosomes are small extracellular lipid vesicles that regulate paracrine interactions between tumor cells and normal stroma, neo‐angiogenesis mediators, and local immune cells, therefore supporting TME maintenance [9]. In addition, these nanovesicles transport several biological molecules such as mRNAs, miRNAs, noncoding RNAs, DNA, lipids, and proteins involved in distant cell signaling pathways, thus laying the bases for metastatic dissemination [13, 14].

The presence of extracellular vesicles has been reported in ascitic fluids and peripheral blood of OC patients; however, the mechanisms by which these extracellular vesicles promote tumor progression in the pre‐metastatic niche have not yet been fully elucidated [15]. Massive ascites are consistent and distinctive features in patients with advanced OC [16]; however, the exact role of malignant ascites in the development of intraperitoneal OC metastases remains unclear, although, if abundant, they are associated with poor prognosis [17]. The ascitic fluid contains tumor cells, lymphocytes, mesothelial cells, macrophages, soluble angiogenic and growth factors, cytokines, chemokines, and extracellular matrix components, contributing to tumor growth and spreading into the peritoneal cavity [18]. At present, therefore, there are many checkpoints related to cancer‐released extracellular vesicles that can be developed to understand better the complex mechanisms of metastatic cancer diffusion and subsequent chemoresistance. Furthermore, the cellular components of ascitic fluids represent an excellent source of tumor tissue to identify prognostic, predictive biomarkers, and molecular profiling analyses [18]. As the tumor cells in the ascites play a crucial role in disease recurrence, a comprehensive understanding of the biological role and clinical relevance of the ascites microenvironment from chemo‐resistant and chemo‐naive patients could be instrumental for effective therapeutic interventions [19]. Besides, specific biomarkers, such as differential expression levels of proteins/RNAs related to the patient’s chemosensitivity status, can be enriched and more stable in ascites‐derived extracellular vesicles.

This study compared the protein profile and the morphometric characteristics of small‐EVs concurrently isolated from the bulk ascitic fluid and ascites‐derived tumor cell cultures. Subsequently, we evaluated the expression of a biomarker panel in the patient's ascites before and after the chemotherapy. We refer to small‐EVs according to size (< 200 nm), as stated by the International Society for Extracellular Vesicles [20].

## 2 Materials and methods

### 2.1 Patients’ cohort

Patients suspected of advanced OC (high‐grade epithelial OC, histological grade G3 confirmed with histological examination, definitive analysis, or frozen section) with bulky stage IIIB‐IIIC to IV disease (FIGO stage classification) afferent at our Institution underwent diagnostic laparoscopy to determine the likelihood of resectability [21], according to the regular clinical practice. Based on the intraoperative clinical evaluation in terms of cytoreduction, following the National Guidelines, patients are subjected to primary debulking surgery (PDS) or neoadjuvant chemotherapy followed by interval debulking surgery (IDS). In addition, the experiments were undertaken with the understanding and written consent of each subject, following approval by the regional ethics committee of Friuli‐Venezia Giulia, Italy (CEUR), protocol number 4829. The study methodologies conformed to the standards set by the Declaration of Helsinki.

### 2.2 Materials

All reagents used to maintain cell cultures and isolate extracellular vesicles were purchased from Thermo Fisher Scientific, Waltham, MA, USA, except otherwise stated. High‐molecular‐weight hyaluronic acid (HMW HA) was kindly provided by Professor Ivan Donati (Department of Life Sciences, University of Trieste, Italy).

### 2.3 Εpithelial ovarian cancer cell (EOC) isolation and characterization

Εpithelial ovarian cancer cell was obtained from ascitic samples by centrifugation at 300 ***g*** for 10 min. Retrieved cells were resuspended in Red Blood Cell Lysis Buffer and washed twice with PBS. Cells were seeded on 6‐well plates (Corning, New York, NY, USA) coated with 50 µg·mL^−1^ of HMW HA. EOC cells were grown at 37 °C, 5% v/v CO2 in human endothelial serum‐free medium (HESFM), containing 20 ng·mL^−1^ b‐FGF, 10 ng·mL^−1^ EGF, 10% fetal bovine serum (FBS), and 1% penicillin/streptomycin [22, 23]. Once EOC cells reached 95% confluency within 3–5 days, they were detached and plated in T75 flasks coated with HMW HA and employed within 1/2 passages.

### 2.4 Immunocytochemical staining

Εpithelial ovarian cancer cells were seeded into eight multichamber slides (Falcon, Corning) at a density of 2 × 10^4^. Following cell attachment, cells were fixed with paraformaldehyde 4% and dried in a laminar flow hood. Hematoxylin and eosin (H&E) staining was performed using an automated stainer instrument (Compass Stainer–Hologic, Marlborough, MA, USA) with Mayer’s hematoxylin and 1% eosin reagents (Leica Biosystem, Buffalo Grove, IL, United States) according to the manufacturer’s instructions. Immunohistochemical staining of EOC fixed cells was performed using BOND‐III Automated IHC Immunostainer (Leica Biosystem). Ki67 (Ki67: Clone MM1 cod.PA0118), MCK (multicytokeratin: clone AE1/AE3 cod. PA0909), and vimentin (clone V9 cod. PA0640) were applied as primary antibodies; staining was developed with the DAB Enhancer utilizing horseradish peroxidase (HRP) conjugated secondary antibodies. All antibodies and reagents were purchased from Leica (Leica Biosystem) and diluted according to the manufacturer’s instructions. All images were acquired by the Cytation 5 cell imaging multimode reader with the objective lens at 20× magnification and processed by gen.5 software (BioTek, Bad Friedrichshall, Germany).

### 2.5 Immunofluorescence staining

Εpithelial ovarian cancer cells were seeded on glass coverslips, and when they reached the 80% confluence, they were fixed with 100% methanol chilled at −20 °C for 5 min. Cells were treated with blocking solution (1% BSA, 22.52 mg·mL^−1^ glycine in PBS+ 0.1% Tween 20) to eliminate unspecific binding for 30 min at room temperature. Cells were incubated with primary anti‐MUC16 antibody (EPSISR23) diluted 1 : 150 overnight at 4 °C and then with the secondary Goat Anti‐Rabbit IgG H&L Alexa Fluor^®^ 488 antibody (ab150077, ABCAM plc Cambridge, UK) diluted 1 : 1000 in 1% BSA for 1 h at RT protecting them from light. The glass coverslips were mounted using ProLong Diamond Antifade Mountant with DAPI, and the images were acquired using Cytation 5 cell imaging multimode reader with objective lens 20× and processed by gen.5 software (BioTek).

### 2.6 Small‐EV isolation

Small‐EVs from ascites were isolated using Total Exosome Isolation Reagent (Invitrogen, Thermo Fisher Scientific, Waltham, MA, USA, CN 4484453), following the protocol specified by the manufacturers. 90% confluent EOC cells were incubated for 24 h with HESFM supplemented with exosome‐depleted fetal bovine serum. Small‐EVs were isolated from the culture medium using Total Exosome Isolation Reagent (Invitrogen, CN 4478359) following the manufacturer’s instructions. Small‐EVs were isolated from two distinct sources. The first source was the bulk fluid ascites, namely the whole ascitic fluid taken from the operating room. The second source was the culture medium of the tumor cells isolated from the ascitic fluid.

### 2.7 Transmission electronic microscopy (TEM)

The *s*ize of small‐EVs was investigated by transmission electron microscopy (TEM), using a Philips CM 100 transmission electron microscope operating at 80 kV, and *s*tandard 3.05 mm copper/Formvar film grids were used (Formvar, 400 mesh, PELCO^®^ grids, Ted Pella, Inc., Redding, CA, USA). Analysis of TEM images to extract the diameter of small‐EVs was performed with the imagej software (Rasband, W.S., ImageJ, U. S. National Institutes of Health, Bethesda, MD, USA, https://imagej.nih.gov/ij/, 1997–2018.). Small‐EV diameter was calculated from different TEM images after manually collecting data corresponding to the vesicle area. The sample preparation and deposition scheme for TEM analysis was performed considering the paper by Rikkert *et al*. [24]. The protocol for the preparation of TEM samples was performed at room temperature and was composed by a fixation step to preserve small‐EV morphology, by the adsorption of small‐EVs to the TEM grid, and by the negative staining with uranyl acetate to enhance the contrast with the background. The sample was placed on the formvar film side of the grid, and the used liquids were filtered using 0.22‐μm RC syringe filters.

### 2.8 Fixation and deposition protocol

A small volume of the sample containing small‐EVs (20 µL) was diluted 1 : 1 with PBS 1× (pH 7.4, ˜ 1.0 mL, Sigma‐Aldrich) and homogenized by vortex stirring. This suspension was then diluted 1 : 1 with a 2% solution of glutaraldehyde (25% v/v; Sigma‐Aldrich) and fixed for 30 min. A volume of 10 μL of this solution was placed on the TEM grid and incubated for 10 min. Excess liquid was removed from one side of the grid using a small piece of blotting paper. Washing of the grid was performed twice by carefully placing the grid (deposition side) on two different drops of Milli‐Q water (100 μL, ˜ 30–60 s each) set on a piece of Parafilm^®^. The excess liquid was removed from the side of the grid with blotting paper. Finally, in a similar way, the grid was located on a 30 μL drop of 1.5% uranyl acetate (w/v) for about 10 s, and the excess solution was removed from one side of the grid by blotting paper.

### 2.9 Dynamic Light Scattering (DLS)

The hydrodynamic diameter distributions of small‐EVs were determined by Dynamic Light Scattering (DLS) measurements with a Malvern Nano ZS instrument equipped with a 633 nm laser diode (Malvern Panalytical Ltd, Malvern, UK). For DLS measurement, 20 µL of small‐EV suspension was dispersed in PBS 1× (pH 7.4, ˜ 1.0 mL, Sigma‐Aldrich, MO, USA) by vortex stirring. The suspension was then transferred in a disposable low volume PMMA cuvette of 1 cm optical path length and was filtered in the same cuvette three times with a 0.22‐µm RC syringe filter (Corning). The hydrodynamic data of the samples were presented, showing the average hydrodynamic diameter (intensity mean), the corresponding volume and number mean diameter values, and the Polydispersion Index (PdI). In the case of a monomodal distribution (Gaussian) calculated through cumulant analysis, PdI = (σ/Zavg)^2^, where σ is the width of the distribution, and Zavg is the average diameter (by intensity) of the particle’s population, respectively. The reported standard deviation (SD) was calculated from the repetition of five measurements of the same sample and was reported to indicate the reproducibility of the DLS measurements.

### 2.10 Surface preparation and sample deposition

All AFM experiments were performed on poly‐L‐lysine (PLL) coated glass coverslips. All reagents were purchased from Sigma‐Aldrich Inc (www.sigmaaldrich.com) unless otherwise stated. Microscopy glass slides (15‐mm‐diameter round coverslips, Menzel Gläser) were cleaned in Piranha solution for 2 h and additionally washed in a sonicator bath (Elmasonic Elma S30H) for 30 min in acetone, followed by 30 min in isopropanol and 30 min in ultrapure water (Millipore Simplicity UV). Glass slides underwent a 5 min of plasma etching (Pelco Easiglow) followed by 30 min in a 0.1 mg·mL^−1^ PLL solution in Borate buffer (pH 8.33) at room temperature. Next, glass slides were thoroughly rinsed with ultrapure water and dried with nitrogen. A 10 μL droplet of the vesicle‐containing solution understudy was deposited on a PLL‐functionalized glass slide, left to adsorb for 10 min at 4 °C, and inserted in the AFM fluid cell without further rinsing. The concentration of each vesicle‐containing solution was adjusted by trial and error in successive depositions to maximize the surface density of individual isolated vesicles and minimize clusters of adjoining vesicles.

### 2.11 AFM imaging and morphometric analysis

Images were collected with an Atomic Force Microscope Multimode VIII equipped with a Nanoscope V (Bruker, Santa Barbara, CA, USA) controller and operated in liquid in ScanAsyst mode. The cantilever chosen for imaging was the SNL‐10 (Bruker) with a nominal spring constant of 0.35 N·m^−1^ and a tip radius of 2 nm. Images were analyzed as described in previous work to describe the sample regarding the size distribution of the small‐EVs and their contact angle, a fingerprint of their mechanical properties [25].

### 2.12 Protein extraction

Total proteins were extracted from small‐EVs using a lysis buffer containing 0.2% Triton X‐100, 50 mm HEPES (pH 7.5), 100 mm NaCl, 1 mm MgCl2, 50 mm NaF, 0.5 mm Na3VO4, 20 mm β‐glycerophosphate, 1 mm phenylmethylsulfonyl fluoride, leupeptin (10 μg·mL^−1^), and aprotinin (10 μg·mL^−1^). Small‐EV lysates were centrifuged at 16 000 ***g*** for 15 min at 4°C, and protein concentration of the supernatants was measured by Bradford assay (Bio‐Rad, Hercules, CA, USA).

### 2.13 Western blotting

Equal protein amounts of extracts prepared from ascites (30 µg) or ascites‐derived tumor cells (50 µg) were analyzed by SDS/polyacrylamide gel electrophoresis and western blotting (WB) using the following antibodies: TSG101 (ABCAM, ab125011), CD63 (ABCAM, ab213090), CD9 (ABCAM, ab2215), antifibronectin antibody (Sigma‐Aldrich, F3648), anti‐TGF‐βI (Sigma‐Aldrich, AV44269), and anti‐PAI‐1 antibody (Sigma‐Aldrich, WH0005054M1). Clarity max Western ECL Substrate (Bio‐Rad) was used to develop the membranes. The kit comprised Peroxidase Buffer and Luminol to develop the immunochemical signal and capture the signal was used a CCD‐camera‐based imager such as the ChemiDoc MP (Bio‐Rad).

### 2.14 Monodimensional gel electrophoresis

60 µg of protein from ascites and 200 µg of protein from cell‐derived small‐EVs were solubilized in Laemly buffer. The protein lysates were separated by 4–20% precast gel (Biorad). After electrophoresis, the gel was fixed with 40% ethanol and 10% acetic acid for 12 h. The gel was then stained with colloidal Coomassie (Sigma) for 48 h, and, after staining, the gel was destained with deionized water. Bands (spots) of interest were excised with a clean scalpel.

### 2.15 In‐gel digestion

Protein in‐gel digestion was performed as previously described by Shevchenko *et al*. [26]. Briefly, gel pieces were dehydrated with acetonitrile; reduction of S‐S protein bridges was obtained with a solution of 10 mm DTT/0.1 m NH4HCO3 and incubation for 30 min at 56 °C. Alkylation was achieved with a solution of 55 mm iodoacetamide/0.1 m NH4HCO3 and incubation in the dark for 20” at room temperature. Gel pieces were next destained, rehydrated, and digested in sequencing grade modified trypsin 12.5 µg·µL^−1^ diluted 1 : 40 in 50 mm NH4HCO3 (Promega Corporation, Madison, WI, USA) overnight at 37 °C. The next day, samples were incubated with 5% formic acid at 37 °C under shaking for 15” and subsequently with acetonitrile at 37 °C under shaking for 15”. The extracts were then dried down in a vacuum centrifuge.

### 2.16 LC‐MS/MS

Peptide samples were purified and concentrated in a porous C18 reversed‐phase resin (Pierce C18 Spin columns, Thermo Fisher). Samples were next resuspended in 0.1% formic acid/5% acetonitrile and analyzed with LC‐MS/MS (Q‐Trap 6500+), while the raw data files were investigated with the Mascot search engine version v.2.6 (Matrix Science, London, UK). As described in an earlier study [27], the spectra were searched against the human section of the UniProt database applying the following parameters: Enzyme specificity was set to trypsin with 1 missed cleavage allowed, and precursor and fragment ions tolerance were 0.6 Da. Carboxymethylcysteine and oxidation of methionine were selected as fixed and variable modifications, respectively. Proteins were considered positive hits if at least 2 unique peptides for each protein were identified with high confidence (FDR ≤ 1%).

### 2.17 Proteome data enrichment and pathway analysis

Gene lists derived from proteomic data underwent KEGG, Gene Ontology (GO), and Reactome (REAC) enrichment analysis using g: Profiler (https://biit.cs.ut.ee/gprofiler/). Venn diagrams were made employing the online tool Bioinformatics & Evolutionary Genomics (http://bioinformatics.psb.ugent.be/webtools/Venn/). A protein–protein interaction network was constructed via the freely available advanced network visualization (web)application inBio Discover™, entering the unique proteins list at the site: https://inbio‐discover.com. The network only showed direct interactions between proteins included in the search proteins (0‐order network). Proteins in the network were shown as gene names at a relevance score of 1. The option “Mark pathway interactions” allowed us to visualize which synergies in the network have supporting evidence from pathway databases (Reactome and WikiPathways) and which ones have supporting evidence only from experimental protein‐protein interaction studies. Interactions with supporting evidence from pathway resources are highlighted in blue.

### 2.18 Statistical analysis

Statistical analyses have been conducted with graphpad prism version 8.0.2 software (GraphPad Software, San Diego, CA, USA). Two‐tailed *t*‐tests were used for protein expression analysis. *P* values < 0.05 have been considered statistically significant.

## 3 Results

### 3.1 Patients’ selection and clinical data

**Fig. 1:**
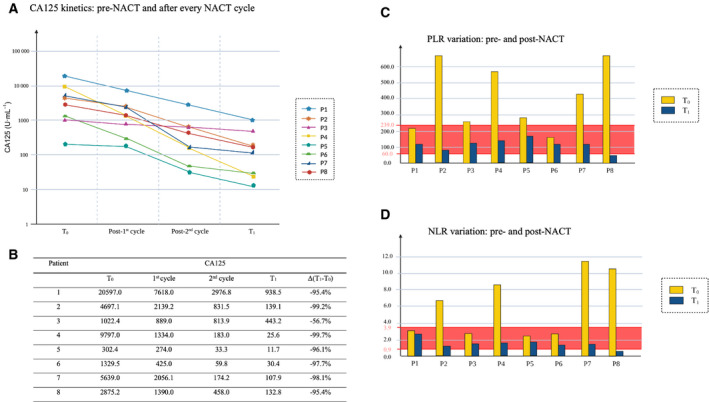
CA125 kinetic and systemic inflammatory response markers. Measurement of different parameters which are considered independent predictors of chemotherapy response. (A) The graph reports changes in CA125 serum levels in the patient cohort throughout chemotherapy. U·mL^−1^: units per milliliters. (B) The table reports values in CA125 serum levels, and their percentage decrease between T0 and T1. T_0_: time of diagnosis before starting neoadjuvant chemotherapy (NACT). T_1_: time of interval debulking surgery (IDS). (C) PLR: platelet‐to‐lymphocyte ratio. (D) NLR: neutrophil‐to‐lymphocyte ratio. The range considered normal for PLR (60.0–239.0) and NLR (0.9–3.9) is highlighted in red.

Eight patients with advanced OC were investigated in this study. Individual clinical data are reported in Table 1 and Fig. 1. A laparoscopic assessment for each patient was performed through a validated laparoscopic scoring algorithm (total predictive index value = PIV) [21, 28]. To assess the clinical response to therapy, we measured different parameters which were considered independent predictors of chemotherapy response by several studies, in two consecutive moments: T_0_, the time of diagnosis before starting neoadjuvant chemotherapy (NACT), and T_1_, the time of interval debulking surgery (IDS), after the third cycle of NACT. CA125 is the most commonly used serum marker in ovarian cancer suspicion and is measured to monitor response to therapy and detect disease recurrence. Platelet‐to‐lymphocyte ratio (PLR) and neutrophil‐to‐lymphocyte ratio (NLR), derived from the absolute platelets, neutrophils and lymphocytes counts of a full blood count, have been previously described as available markers of the systemic inflammatory response [29]. In particular, the kinetic of these markers was considered to be related to the NACT response. We observed a progressive reduction in CA125 after each dose of chemotherapy drugs (Fig. 1A), and the percentage decrease between T_0_ and T_1_ ranged from 56.7% to 99.2% (Fig. 1B). However, only three patients achieved the normalization of the CA125 value (0–35 units·mL^−1^). The same has been noted considering PLR and NLR; respectively; the percentage decrease was between 28% and 91%, and 17% and 95%. Nevertheless, in this case, all eight patients reached the range considered normal both for PLR (60.0–239.0) and NLR (0.9–3.9) (Fig. 1C,D).

**Table 1:** Patient selection and clinical data.

Pt	CA125 (t_0_)	CA125 (t_1_)	Ascites mL (t_0_)	Ascites mL (t_1_)	PIV (t_0_)	PIV (t_1_)	Histology
1	20 597	938.5	200	< 100 mL	8		HGSOC FIGO IV
2	4697.1	139.1	4900	0	8	4	HGSOC FIGO IIIC
3	1022.4	443.2	1800	300	8	2	HGSOC FIGO IIIC
4	9797	25.6	7000	< 100 mL	10	4	HGSOC FIGO IIIC
5	302.4	11.7	2000	0	10	4	HGSOC FIGO IV
6	1329.5	30.4	< 100 mL	0	8	2	HGSOC FIGO IIIC
7	5639	107.9	4000	< 100 mL	8	6	HGSOC FIGO IIIC
8	2875.2	132.8	3700	< 100 mL	8	6	HGSOC FIGO IIIC

HGSOC, high‐grade serous ovarian cancer; PIV, predictive index value (Fagotti score); PLR, platelet‐to‐lymphocyte ratio; t_0_, diagnosis; t_1_, post‐neoadjuvant chemotherapy (post‐NACT).

### 3.2 Multicytokeratin, Ki67, Mucin‐16, and vimentin are highly expressed in EOC cells

**Fig. 2:**
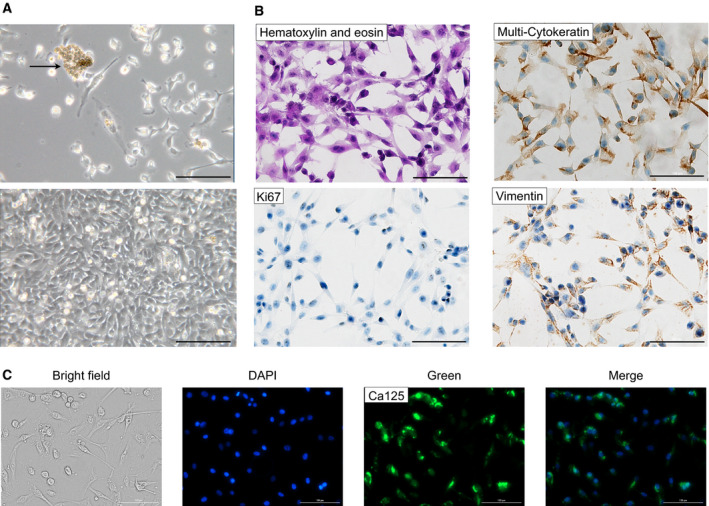
Morphology and identification of ascites‐derived epithelial ovarian cancer (EOC) cells. (A) Representative phase‐contrast images of EOC cells. In the upper image: cells after initial isolation, grape‐like cluster (arrow), and single EOC cells. In the lower image: a confluent monolayer of EOC cells expanded in culture. Note the typical epithelial cobblestone morphology and tight cell‐to‐cell junction. (B) Subsequent cytological analysis. Immunochemistry of EOC cells showed expression of epithelial marker multicytokeratin, mesenchymal marker, vimentin, and cell proliferation marker Ki67. (C) Immunofluorescence staining of EOC cells revealed the expression of the Mucin‐16 tumor marker (CA125). Brightfield, nucleus staining with blu DAPI, Mucin‐16 marker staining with green Alexa Fluor^®^ 488, and merge image (DAPI+ Alexa Fluor^®^ 488) are reported. Bar = 100 μm. For each set of analyses, representative results out of three independent experiments are shown.

Epithelial ovarian cancer cells were initially seeded in six‐multiwell plates until they reached 95% confluence. Adherent cells were in balance during initial plating with nonadherent grape‐like cell clusters (Fig. 2A, upper panel). Cells were then transferred in a T75 flask and were grown to confluence for 3–5 days. Cells grew in swirl‐like clusters, and when they reached 100% confluence, they formed a monolayer with the typical ‘cobblestone’ epithelial morphology without contaminations of stromal cells shown in Fig. 2A lower panel [23]. The phenotypic characteristics of EOC cells were confirmed, seeding 2 × 10^4^ cells on chamber slides to perform the immune‐cytochemical analyses. In Fig. 2B, H&E staining shows the morphology of growing 100% confluent EOC cells, characterized by frequent positivity for Ki67, a marker of proliferation and aggressiveness clinically employed to grade several human cancers. Multicytokeratin staining was evenly distributed in EOC cells' cytoplasm and confirmed the epithelial origin of ascites‐derived EOC cells. Immunostaining for vimentin, a specific cytoskeletal protein, showed a high cytoplasmic expression, suggesting that these cells underwent epithelial–mesenchymal transition acquiring a neoplastic phenotype [30]. The expression of Mucin‐16, the gold standard marker in OC, confirmed that EOC cells isolated by the patients’ ascites own a tumor phenotype with green staining on the cell membrane (Fig. 2C).

### 3.3 Exosome derived from bulk fluid ascites and tumor cells display a high expression of CD9, TSG101 and CD63 and present distinctive morphometric properties

#### 3.3.1 Atomic Force Microscopy (AFM) imaging and quantitative morphometry of small‐EVs

**Fig. 3:**
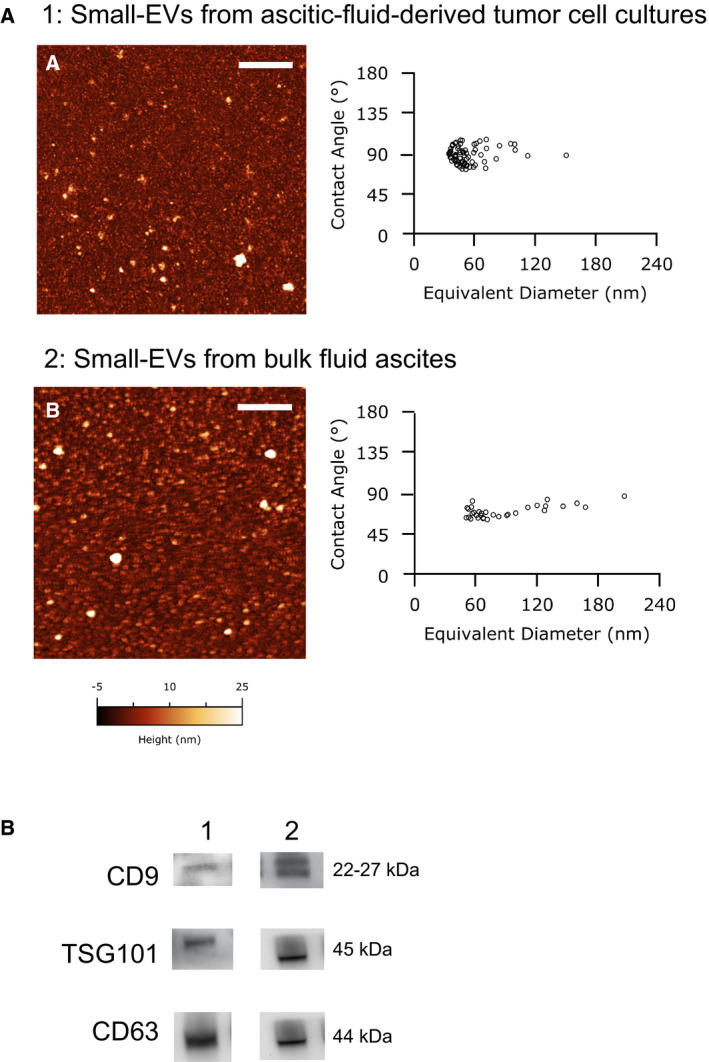
Characterization of small‐EVs isolated from human ovarian ascites fluid. (A) Representative liquid Atomic Force Microscopy (AFM) micrographs of small‐EVs from ascites‐derived tumor cells (A) and small‐EVs from bulk fluid ascites (B), the length of both scale bars is 1 µm. On the right: Contact Angle vs Diameter scatterplot of small‐EVs. Each circle represents one individual exosome as measured via AFM imaging in liquid. (B) CD63, CD9, and TSG101 were detected by western blot in small‐EVs extracted from ascites‐derived tumor cell cultures (1) and bulk fluid ascites small‐EVs (2). For each set of analyses, representative results out of three independent experiments are shown.

Small‐EVs isolated from two distinct sources (the bulk fluid ascites, namely the whole ascitic fluid taken from the operating room, and the culture medium of the tumor cells isolated from the ascitic fluid) were analyzed via the AFM‐based high‐throughput nanomechanical screening method described elsewhere [25]. Briefly, this method entailed depositing vesicles on a substrate with a controlled surface charge density. Upon their interaction with the substrate, vesicles adopted a spherical cap shape characterized by a specific vesicle/surface contact angle (CA). A quantitative correlation existed between a vesicle’s CA and its mechanical characteristics, with softer vesicles assuming shallow (i.e., more oblate) shapes characterized by lower CAs than stiffer ones. CAs of individual vesicles and their size could be accurately measured via AFM imaging performed in liquid. Figure 3A shows representative AFM micrographs of small‐EVs isolated from either bulk fluid ascites or ascitic fluid‐derived tumor cells. A first qualitative inspection of AFM images revealed that both samples only contained nanometric globular objects with no other significant contaminants. Quantitative AFM morphometry was then performed, as described above, and the diameter vs. CA plots were reported in the left panels. A single horizontally elongated cluster of points emerged in both cases, indicating that all globular objects in the sample have a characteristic stiffness conserved across the whole range of diameters. This type of nanomechanical behavior was previously linked to lipid vesicles [25]; if present, any nonvesicular contaminants would appear here as a distinct cluster characterized by a broader range of CA [31]. These observations, considered together, suggested that both samples only contained nanosized small‐EVs with high purity. Small‐EVs derived from bulk fluid ascites were found to be larger (average diameter = 90 nm, standard deviation = 40 nm, *N* = 33) and softer (average CA = 70°, standard deviation = 7°, *N* = 33) than small‐EVs obtained from ascitic fluid‐derived tumor cells (average diameter = 55 nm, standard deviation = 20 nm; average CA = 89°, standard deviation = 9°; *N* = 75), and data are also presented in Table 2. This observation implied that the two samples contained different types of small‐EVs.

**Table 2:** AFM, TEM, and DLS data for small‐EVs in ascites fluid and ascitic fluid‐derived tumor cells.

Sample	dAFM ± SD (nm)	dTEM ± SD (nm)	Average hydrodynamic diameter (volume mean) ± SD (nm)	Average hydrodynamic diameter (intensity mean) ± SD (nm)	Polydispersion Index (PDI)
1. Small‐EVs from bulk fluid ascites	90 ± 40	20 ± 2	26 ± 4	54 ± 2	0.52
2. Small‐EVs from ascites‐derived tumor cells	55 ± 20	43 ± 14	78 ± 6	130 ± 20	0.46

#### 3.3.2 TEM and Dynamic Light Scattering (DLS)

TEM images of small‐EVs in the samples from ascites fluid and ascitic fluid‐derived tumor cells were obtained with a protocol presenting a fixation step performed in solution to preserve small‐EV morphology and the negative staining step on the TEM grid obtained using a diluted uranyl acetate solution. This final step was necessary to enhance the contrast of the small‐EVs with the background. A representative TEM image of EVs from ascites fluid and their diameter distribution are shown in Fig. S1A. This sample presented a monodisperse small‐EV distribution, with an average diameter = 20 nm, and an SD = 2 nm (*N* = 330). On the other hand, TEM analysis of small‐EVs deriving from ascitic fluid‐derived tumor cells showed a more polydisperse distribution (Fig. S1B), with an average diameter = 43 nm, and an SD =14 nm (*N* = 280). DLS measurements of samples containing small‐EVs from ascites fluid and ascitic fluid‐derived tumor cells were performed in PBS 1× (pH 7.4, 25 °C) and confirmed. The results obtained from DLS investigation (average hydrodynamic diameter, intensity mean) showed that the average hydrodynamic diameter of small‐EVs' in the two samples was quite different, with the presence of multiple hydrodynamic size distributions and consequently relatively high Polydispersion Index (PDI) (Table 2). However, the corresponding DLS hydrodynamic size distributions represented by volume better represent the principal populations of the small‐EVs in the samples (Fig. S2). The DLS volume means hydrodynamic diameters, and the diameters obtained by TEM analysis showed in Table 2 are quite comparable. Larger dimension was imputable to the measurement of the hydrodynamic diameter (comprising the solvation layer and/or protein corona around the small‐EVs) and to the strong tendency of the DLS technique to overweight the contribution of aggregated particles within the samples.

#### 3.3.3 Expression of CD9, TSG101, and CD63

Preparations were lysed and subjected to western blot analyses employing the specific markers CD9, TSG101, and CD63, to verify the presence of small‐EVs. Figure 3B shows the expression of the small‐EV‐specific markers in representative preparations.

### 3.4 Proteomic profiling of the ascites‐derived small‐EVs

#### 3.4.1 Bulk fluid ascites’ derived small‐EVs

**Fig. 4:**
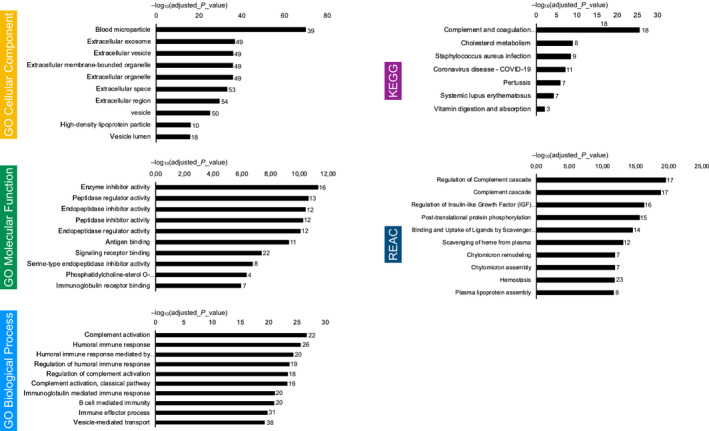
Proteomic profiling of the small‐EVs from bulk fluid ascites. Gene lists derived from proteomics data (*n* = 8) underwent KEGG, Gene Ontology (GO), and Reactome (REAC) enrichment analysis using g: Profiler. Negative log10 of adjusted *P*‐value and intersections size are reported in graphs.

In the present study, one‐dimensional gel electrophoresis and shotgun proteomic analyses identified a total of 104 unique proteins in bulk fluid ascites’ derived small‐EVs (Table S1). Analysis of proteomic data was performed to identify activated pathways or pathway modules, and through the Gene Ontology (GO) representation, to assess the biological significance of the proteomic results (Fig. 4) [32]. GO‐Cellular Component (GO‐CC) analysis showed that most of the proteins were concentrated in blood microparticles and extracellular exosome/vesicle/organelle, further confirming the efficient small‐EV isolation. GO‐Molecular Function (GO‐MF) analysis showed that proteins were mainly related to peptidase regulator/inhibitor activity, endopeptidase regulator/inhibitor activity, signaling receptor binding, immunoglobulin receptor binding, and antigen‐binding. GO‐Biological Process (GO‐BP) analyses revealed that proteins were significantly enriched in biological processes, including humoral immune response, complement activation, defense response, vesicle‐mediated transport, endocytosis, B‐cell‐mediated immunity, and innate immune response. In the KEGG pathway analyses, the proteins of the complement and coagulation cascade were significantly enriched. We also mapped the proteins to Reactome pathways (REAC), which highlighted the enrichment of regulation of complement cascade, regulation of insulin‐like growth factor (IGF) transport, and post‐translational protein phosphorylation among the most significant.

#### 3.4.2 Small‐EVs from ascites‐derived tumor cells

**Fig. 5:**
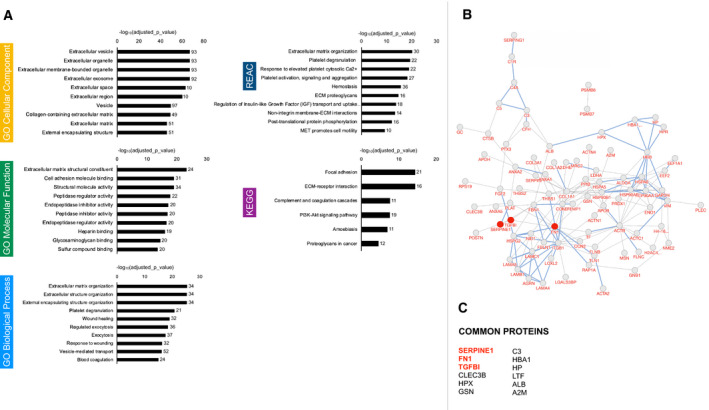
Proteomic profiling of the small‐EVs from ascites‐derived tumor cells. (A) Gene lists derived from proteomics data (*n* = 8) underwent KEGG, Gene Ontology (GO), and Reactome (REAC) enrichment analysis using g:Profiler. Negative log10 of adjusted *P*‐value and intersections size are reported in graphs. (B) Protein–protein interaction network visualized by BioDiscover^TM^. Interactions with supporting evidence from pathway resources are highlighted in blue. Fibronectin, TGFβI, and PAI‐1 are highlighted in red. (C) 12 proteins were identified that were common to every EV sample.

A total of 109 unique proteins were identified in the small‐EVs from supernatants of ascitic fluid‐derived tumor cells (Table S1). Analysis of pathways and GO representation are shown in Fig. 5. GO‐CC analysis unveiled that most of the proteins were concentrated in extracellular exosome/vesicle/organelle, further confirming the efficient small‐EV isolation, extracellular matrix, and extracellular region/space. GO‐MF analysis showed that proteins were mainly related to extracellular matrix structural constituent, cell adhesion molecule binding, structural molecule activity, peptidase regulator/inhibitor activity, endopeptidase regulator/inhibitor activity, and glycosaminoglycan binding. GO‐BP analyses proved that proteins were significantly enriched in biological processes, including extracellular matrix/structure organization, platelet degranulation, vesicle‐mediated transport, and exocytosis. Of interest, in the KEGG pathway analysis, the proteins were significantly enriched in focal adhesion, ECM‐receptor interaction, complement and coagulation cascade, and PI3K‐Akt signaling pathway. We also mapped the proteins to Reactome pathways (REAC), which highlighted the enrichment of extracellular matrix organization, platelet degranulation, response to elevated platelet cytosolic Ca2+, platelet activation/signaling/aggregation, post‐translational protein phosphorylation, regulation of insulin‐like growth factor (IGF) transport, ECM proteoglycans, and nonintegrin membrane–ECM interactions. Protein–protein interactions analysis results (Fig. 5B) attested that extensive and complex interactions occurred in the unique proteins we found in small‐EVs derived from tumor cell cultures.

### 3.5 Differential protein expression in small‐EVs samples taken before and after chemotherapy

The data emerging from the bioinformatics analysis indicated a strong involvement of vesicular proteins derived from cultured tumor cells in the dynamics of the ECM, and we wondered if the expression of these proteins could change throughout chemotherapy. Therefore, we selected three proteins (see Fig. 5B, highlighted in red) among the 12 common proteins detected in all the samples analyzed, shown in Fig. 5C. We quantified their expression longitudinally in three patients taking samples before and after chemotherapy. Fibronectin plays a prominent role in mediating ECM dynamics because of its high abundance and interaction with cellular components. We decided to evaluate fibronectin expression based on the data already present in the literature describing its critical role in metastatic processes and as a potential target for metastasis prevention and inhibition [33, 34, 35]. Of interest, previous data by Kenny *et al*. [34] suggest that maintenance therapy aimed at preventing the interaction of fibronectin with the fibronectin receptor α5β1 integrin after the end of adjuvant chemotherapy might delay tumor recurrence and should be considered for further clinical testing. The same authors underline that the availability of fibronectin to cancer cells is a molecular factor that is critical for metastasis. Functionally, fibronectin induces tumor cell adhesion, proliferation, and invasion and promotes spheroid formation and adhesion [22, 34]. Moreover, increased expression of fibronectin, a key component of the ECM, has been detected in OC metastases compared with the primary ovarian tumor [36].

Several signaling cascades have been found to use EVs for signaling in the tumor–stroma interaction. These include TGF‐β signaling [37]. TGFβI is an ECM protein functionally linked to fibronectin [38], as it serves as a linker protein that mediates integrin binding to extracellular proteins, such as fibronectin, collagen, and laminins [39]. Epithelial OC cells have the potential to switch between epithelial and mesenchymal states during metastasis, and previous findings showed that the TGFβI signaling significantly impacts epithelial‐to‐mesenchymal transition and promotes the malignant potential of OC spheroid cells [38].

PAI‐1 is a tumor‐promoting factor that accelerates the process of peritoneal metastasis [40]. PAI‐1 correlates with peritoneal metastasis in ovarian cancer patients and indicates a poor prognosis.

**Fig. 6:**
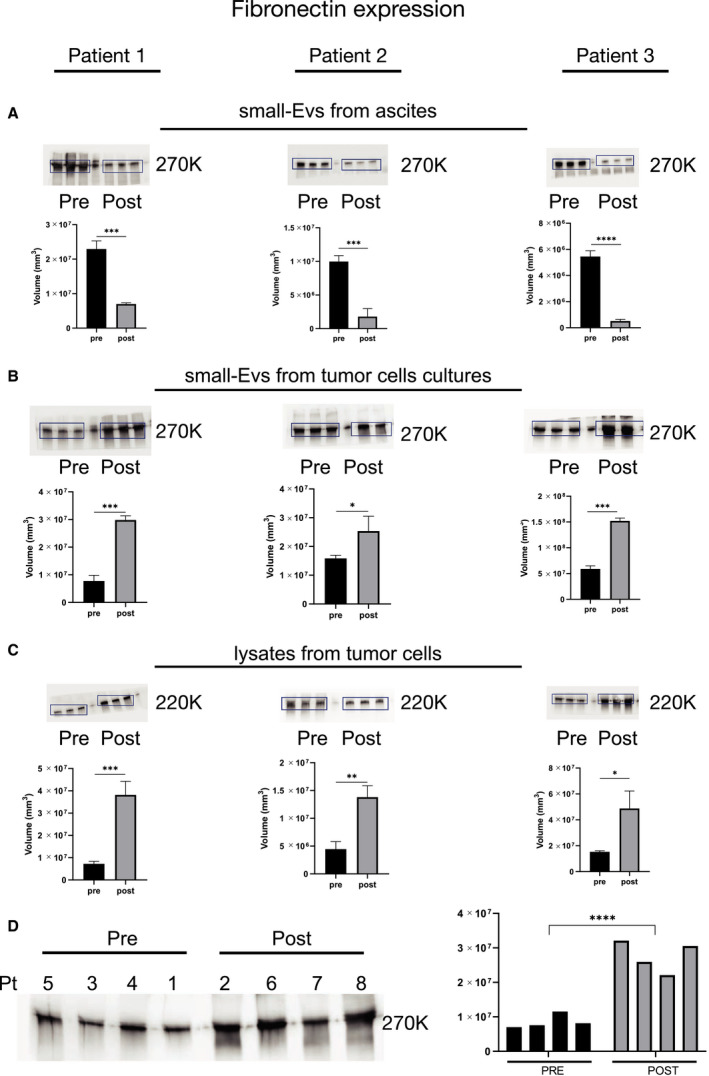
Differential fibronectin expression in small‐EV samples taken before and after chemotherapy. Protein extracts (30 µg) prepared from small‐EVs isolated from bulk fluid ascites (A), small‐EVs isolated from ascites tumor cell cultures (B), and ascites tumor cells lysates (C) of patients 1, 2, and 3 were loaded on a 4–12% SDS/PAGE followed by western blot using antibodies against fibronectin. Two‐tailed *t*‐tests were used for protein expression analysis. Data are reported as means ± SD of one experiment performed in triplicate. *P < 0.05, **P < 0.01, ***P < 0.001, ****P < 0.0001. (D) Analysis of the small‐EVs isolated from ascites tumor cell cultures on the cohort of patients divided into two groups: the patients who did not receive chemotherapy at the time of sampling and the patients who underwent chemotherapy. ****P < 0.0001. The patient’s number corresponds to Table 1.

The fibronectin expression was analyzed in small‐EVs isolated from ascites and ascitic fluid‐derived tumor cells in three patients before and after chemotherapy (Fig. 6). In all three patients (see patients 3, 4, and 8 in Table 1), ascites was extensive at presentation, and neoadjuvant chemotherapy was employed to reduce the patient's tumor burden and to decrease postoperative morbidity at the time of the interval debulking. Two ascites samples were collected during laparoscopic surgery: the first sample at the exploratory laparoscopy performed for diagnostic purposes and the second sample after the completion of neoadjuvant chemotherapy at the time of the interval debulking (three months after the first sample).

**Fig. 7:**
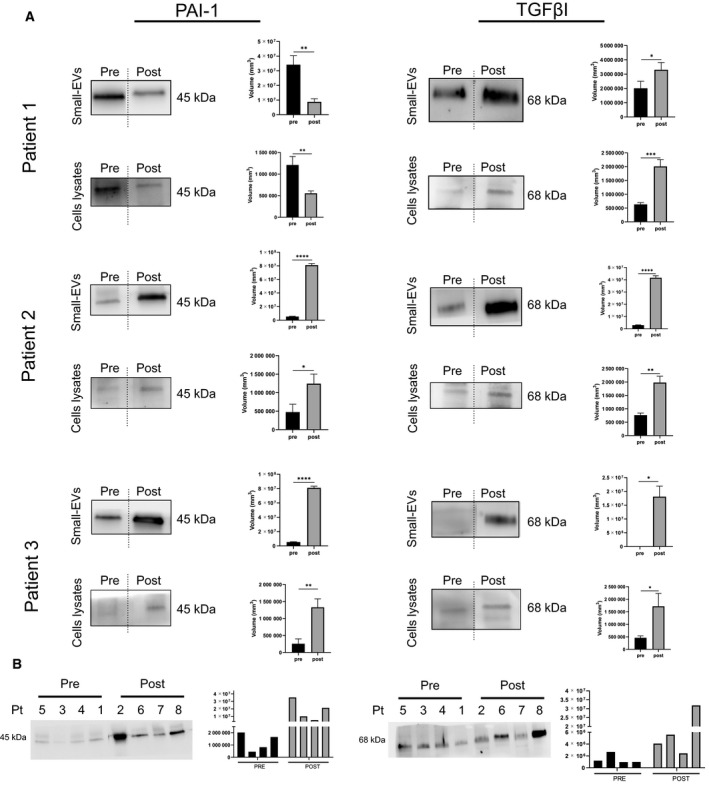
Differential TGFβI and PAI‐1 expression in small‐EV samples taken before and after chemotherapy. (A) Protein extracts (30 µg) prepared from small‐EVs isolated from bulk fluid ascites, small‐EVs isolated from ascites tumor cell cultures, and ascites tumor cell lysates of patients 1, 2, and 3 were loaded on a 4–12% SDS/PAGE followed by western blot using antibodies against TGFβI and PAI‐1. Two‐tailed t‐tests were used for protein expression analysis. Data are reported as means ± SD of one experiment performed in triplicate. *P < 0.05, **P < 0.01, ***P < 0.001, ****P < 0.0001. (B) Analysis of the small‐EVs isolated from ascites tumor cell cultures on the cohort of patients divided into two groups: the patients who did not receive chemotherapy at the time of sampling and the patients who underwent chemotherapy. The patient’s number corresponds to Table 1.

Interestingly, the western blot analyses indicated that the fibronectin expression significantly decreased in small‐EVs from bulk ascitic fluid after neoadjuvant chemotherapy (Fig. 6A), whereas it significantly increased in small‐EVs derived from tumor cell cultures (Fig. 6B). To confirm that the analyzed protein was enriched in small‐EVs, source material lysates were also analyzed by western blot (Fig. 6C). We then analyzed fibronectin expression on a randomly selected subset of small‐EVs isolated from ascites cell cultures, four samples derived from patients in prechemotherapy and four samples from patients who underwent chemotherapy, to determine whether there was an overall trend across the cohort (Fig. 6D). Consistently, we observed a difference in the fibronectin expression between the two groups of samples, with an increased expression in patients who underwent chemotherapy (mean band intensity increase > 200%, *P* = 0.00025). Concurrently, since transforming growth factor‐β‐I (TGFβI) and plasminogen activator inhibitor 1 (PAI‐1) play a pivotal role in modulating ECM expression and remodeling and were identified in all the small‐EVs from tumor cells previously analyzed, we further studied their expression pre‐ and postchemotherapy in the three patients who were followed longitudinally (Fig. 7A). Consistent with fibronectin results, after chemotherapy, we observed an increased expression of TGFβI in the tumor cell‐derived small‐EVs of all three patients. PAI‐1 increased in patients 2 and 3 upon chemotherapy, whereas it decreased in patient 1. We then analyzed TGFβI and PAI‐1 expression on the small‐EVs derived from tumor cell cultures on eight samples randomly selected from patients in pre‐ and postchemotherapy, showing an increased expression in patients who underwent chemotherapy (Fig. 7B). The uncropped full‐length pictures of western blot membranes are shown in Fig. S3.

## 4 Discussion

Malignant ascites‐derived EVs of OC patients arise from a dynamic range of tumor and nontumor cellular populations, particularly cells of the immune system and the tumor stroma, forming a complex microenvironment [41]. However, the biochemical interplay between these cellular populations, how they contribute to the molecular tumor heterogeneity (i.e., different phenotypes, gene expression patterns, and proliferation potentials), and the interaction with the microenvironment remain enigmatic, particularly from the perspective of the tumor proteome [41]. Furthermore, proteomic analysis of complex fluids such as ascites is hampered by the challenge of detecting low‐abundant proteins, while exceedingly highly abundant proteins may not detect those of interest. Concerning the clinical studies on the small‐EVs isolated from OC ascites reported in the literature, they all relied on the total pool of small‐EVs [42, 43, 44], thus lacking a specific signature analysis related to small‐EVs derived from tumor cells. In this respect, our results demonstrated that the characterization of small‐EVs derived from tumor cells short‐term cultures provided information on the extracellular matrix dynamics that were not obtainable from the bulk ascites' analyses. We could outline that essential and extensively studied biomarkers such as fibronectin exhibited opposite expression patterns in small‐EVs in response to chemotherapy when measured in bulk ascites or cultures of patients' tumor cells. This issue indicated that the analysis of the bulk fluid ascites‐derived small‐EVs could be misleading when looking at signatures related to tumor progression and aggressiveness. On the other hand, the decrease in fibronectin in bulk fluid ascites was consistent with some clinical parameters used as a standard of reference to assess therapy response. In particular, CA125 kinetic and systemic inflammatory response markers (PLR and NLR) suggested improving the tumor burden and inflammation after chemotherapy in all the patients. Therefore, to investigate the role of floating tumor cells in ascites and their ability to metastasize, leading to disease recurrence, it was necessary to rely on a specific approach directed at the tumor itself. There is a growing body of evidence that OC cells must breach the mesothelium of peritoneal organs to adhere to the underlying ECM and initiate metastatic growth. Of interest, previous data suggested that the acquisition of a mesenchymal–mesothelial phenotype promotes cancer cell adhesion to the ECM of peritoneal organs and OC progression [45].

We acknowledge the complexity of cancer metastasis and the multiscale nature of its progression, which call for a systems approach to understanding the mechanism by which signaling components present in the obtained EVs promote metastatic tumor spread and chemoresistance. We believe that our tumor cell isolation workflow can assist in designing future mechanistic research focused on examining the ascites‐derived EVs. Our bioinformatic analysis on protein profile identified several genes that potentially serve critical roles in the metastatic process via the ‘focal adhesion’, ‘ECM–receptor interaction’, and ‘ECM proteoglycans’ KEGG pathways, suggesting that tumor cell’s small‐EVs may promote the migration of ovarian cancer by mediating the ECM–receptor interaction.

To date, tumor chemosensitivity is evaluated with a histological examination of the *omentum*, according to the CRS system [46]. However, the limit of the CRS system is that it provides us with a static image at a point in time while predicting and monitoring response to therapy and disease progression are essential due to changes at the molecular level in response to treatment. Here, we revealed that the presence of ascites provides an opportunity to study the tumor evolution and investigate how tumor cell’s small‐EV profiles changed during chemotherapy by sampling ascites before and after the therapy mentioned above [18, 47]. We observed that ascites‐derived tumor cell’s small‐EVs isolated after neoadjuvant chemotherapy displayed a differential expression of fibronectin, TGFβI, and PAI‐1, proteins involved in the ECM remodeling. Thus, the modulation of the small‐EVs proteins might occur due to tumor reshaping induced by chemotherapy and could be conducive to seed distant organs. A body of evidence data supports the notion that TGFβI plays a pivotal role in the crosstalk between cells and the surrounding ECM [48]. PAI‐1 is involved in cancer invasion and metastasis by remodeling the ECM through the plasmin‐mediated matrix metalloproteinase activation. PAI‐1 is also a component of the ECM, where it binds to vitronectin.

Among the primary finding of our study, by applying a recently developed high‐throughput AFM‐based mechanical characterization method, which analyses the contact angle (a parameter related to the deformation extent) of a vesicle once adsorbed on a substrate, we demonstrated that small‐EVs derived from the two sources, bulk fluid ascites and tumor cells short‐term cultures, showed a markedly different stiffness. Compared to classical AFM‐based Force Spectroscopy (AFM‐FS), which represents one of the most widely used techniques for the mechanical characterization of lipid nanovesicles [49], the herein employed mechanical characterization allows obtaining information on vesicle mechanics just exploiting AFM imaging; this results in larger number of vesicles being sampled at the same time, together with a less challenging data analysis [50, 51, 52]. Moreover, in the original work, Ridolfi *et al*. [25] derive a linear relationship between the contact angle and vesicle stiffness, measured by AFM‐FS, hence making the former, an appropriate descriptor of vesicle mechanical properties. This result supports findings from recent mechanical studies on vesicles, which found that the small‐EV mechanical characteristics might be tuned for optimal penetration in the extracellular matrix, which requires stiffness values able to maximize both the diffusion velocity of the vesicles in the organism and their deformability upon adhesion [49, 53, 54]. Also, we could observe that the bulk ascitic fluid small‐EVs express proteins predominantly involved in the complement and coagulation cascade. Differently, proteins of tumor‐derived small‐EVs, beyond their involvement in complement and immune system homeostasis, play a significant role in ECM deposition and remodeling. The pre‐metastatic niche concept arises from experimental evidence that suggests that the tumor preconditions specific organ sites for subsequent metastatic seedings through tumor‐derived factors, thus allowing the interplay between tumor cells and their microenvironment [9, 55, 56]. Remarkably, a recent study indicated that tumor‐secreted small‐EVs could induce the metastatic propensity of tumor cells that could not previously metastasize specific organ sites [57]. Consistently, the clinical feature of the patients investigated here revealed an intense aggressiveness of the pathology that manifested itself with a relapse or with a lack of response to therapy, such as not allowing an interval surgery based on Fagotti score.

Finally, the bioinformatic analysis of the small‐EV proteins identified on the patient's tumor cell cultures suggests that these proteins may strongly impact platelets' processes. In particular, we found 27 proteins involved in platelet activation, signaling, aggregation, and 22 proteins involved in platelet degranulation and response to elevated platelet cytosolic Ca2+. While there is no doubt that platelet has a role in the inflammatory processes in the ascites, emerging evidence, including our results, suggests that tumor‐derived small‐EVs can play an active role in the interplay between platelets and cancer cells. Thus, in addition to a prominent role in paraneoplastic thrombocytosis, platelets can play a crucial role in tumor cell invasion, extravasation, and protection from the host immune system through bidirectional communication with tumor cells [58, 59]. However, the exact mechanisms regulating the promotion of peritoneal metastasis by platelets through the ascites are not fully elucidated, and small‐EVs might be pivotal in this context.

## 5 Conclusions

A comprehensive characterization of nature and the components of the small‐EVs released by ovarian cancer cells is needed to understand how metabolic alterations affect the pre‐metastatic niche formation and the therapeutic response. Here, we showed that sampling ascites before and after chemotherapy allows characterizing small‐EVs released by tumor cells, reflecting their biological status. Furthermore, since predicting and monitoring response to therapy and disease progression is essential due to changes at the molecular level in response to treatment over time, we believe that this study on small‐EVs released by tumor cells helps drive assumptions about how best to tackle the dynamics of extracellular matrix. Our tumor cell isolation workflow can assist in designing future mechanistic research focused on examining the ascites‐derived EVs. In particular, our findings indicate that an extensively studied biomarker such as fibronectin exhibits opposite expression patterns in small‐EVs in response to chemotherapy when measured in bulk ascites on the total pool of small‐EVs or specifically in the cultures of patients' tumor cells. Moreover, we observed that tumor‐derived small‐EVs isolated after neoadjuvant chemotherapy showed increased TGFβI, PAI‐1, and fibronectin expression. These findings highlight an ascites cell isolation workflow's importance in investigating the treatment‐induced cancer adaption processes.

## Conflict of interest

The authors declare no conflict of interest.

## Author contributions

BB conceived, designed and performed the experiments on primary cultures, analyzed the data. MA performed the experiments on protein profiling and western blot, and analyzed the data. ER performed the TEM experiments and analyzed the data. FV, MB, and AR performed the AFM experiments and analyzed the data. BU analyzed the mass spectrometry data and provided technical and materials support. RA performed mass spectrometry analysis. GDL, FR, FB, CR, and GR provided patients’ material and characteristics, and analyzed clinical data. SB originally conceptualized, designed the study, performed the bioinformatics analysis, wrote the manuscript, and generated final figures. All authors have read and approved the final manuscript.

## Supporting information


**Fig. S1**. TEM image and size distribution of small‐EVs.

**Fig. S2**. DLS size distribution by volume of small‐EVs.

**Fig. S3**. Uncropped full‐length pictures of Western blot membranes.

**Table S1**. List of unique proteins found in small‐EVs.

## Data Availability

All data generated or analyzed during this study are included in this published article and its supplementary information files.
