# Supplementary material for: Small extracellular vesicles from malignant ascites of patients with advanced ovarian cancer provide insights into the dynamics of the extracellular matrix
Source: Mol Oncol. 2021 Oct 27;15(12):3596–614. doi: 10.1002/1878-0261.13110 (PMC8637559; doi:10.1002/1878-0261.13110)
Supplement: Supplementary file 2 — Fig. S2. DLS size distribution by volume of small‐EVs. [file MOL2-15-3596-s004.pdf]

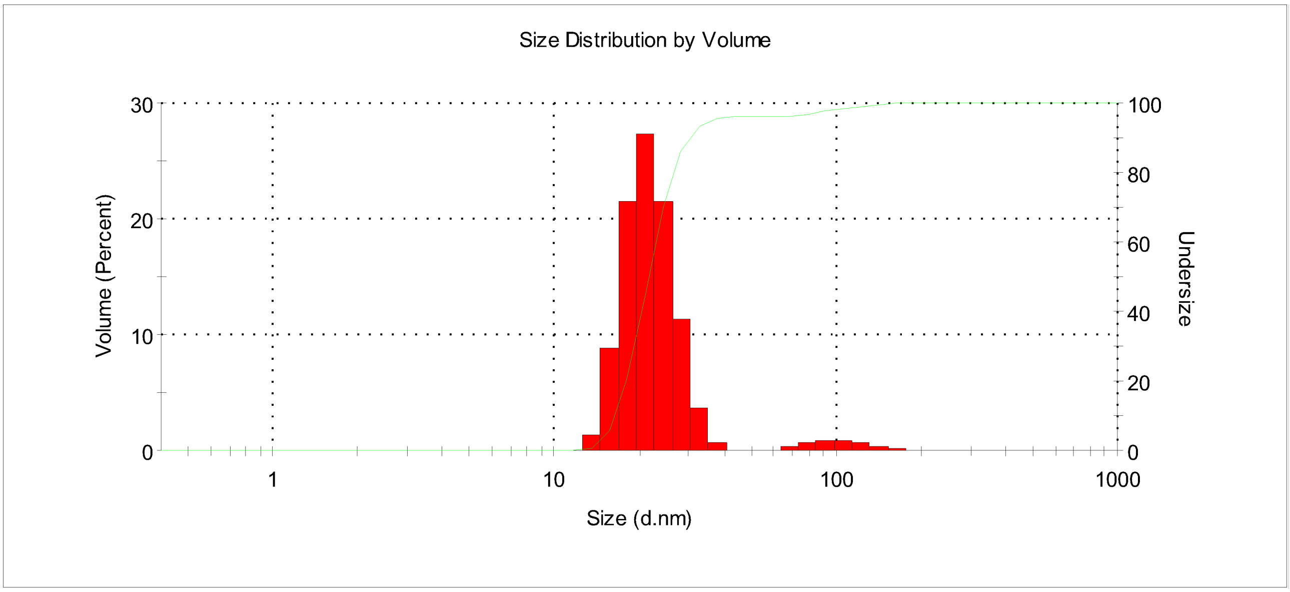

Size distribution by number of small-EVs from bulk fluid ascites (PBS 1X, pH 7.4, 25°C)

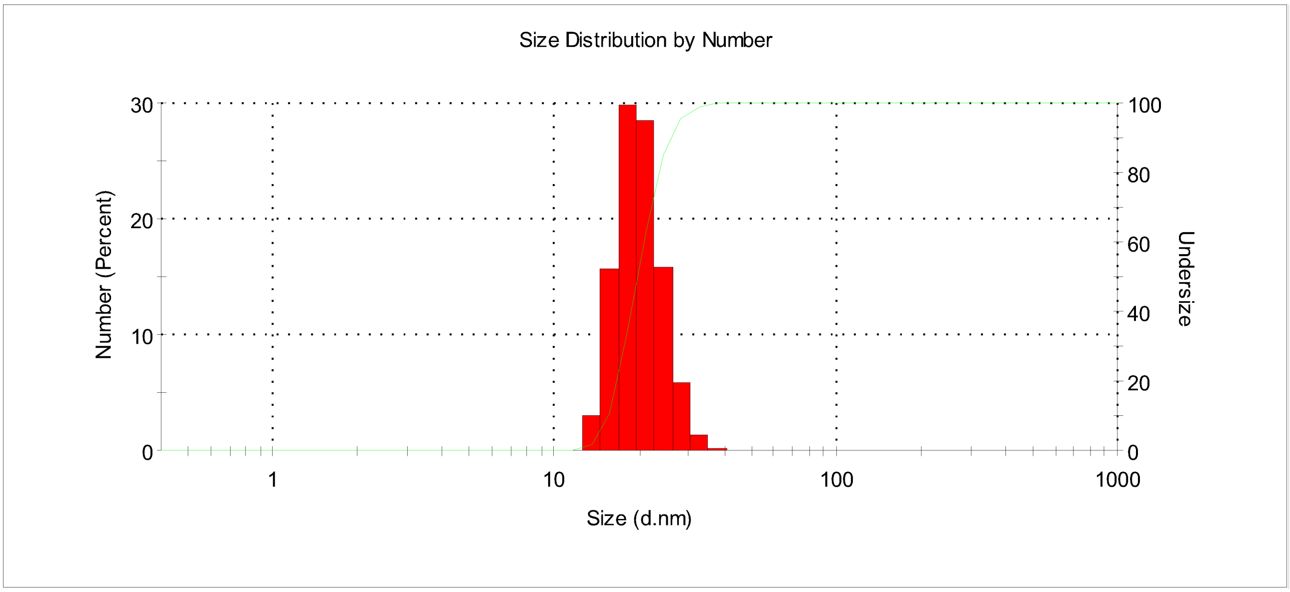

Size distribution by volume of small-EVs from bulk fluid ascites (PBS 1X, pH 7.4, 25°C)

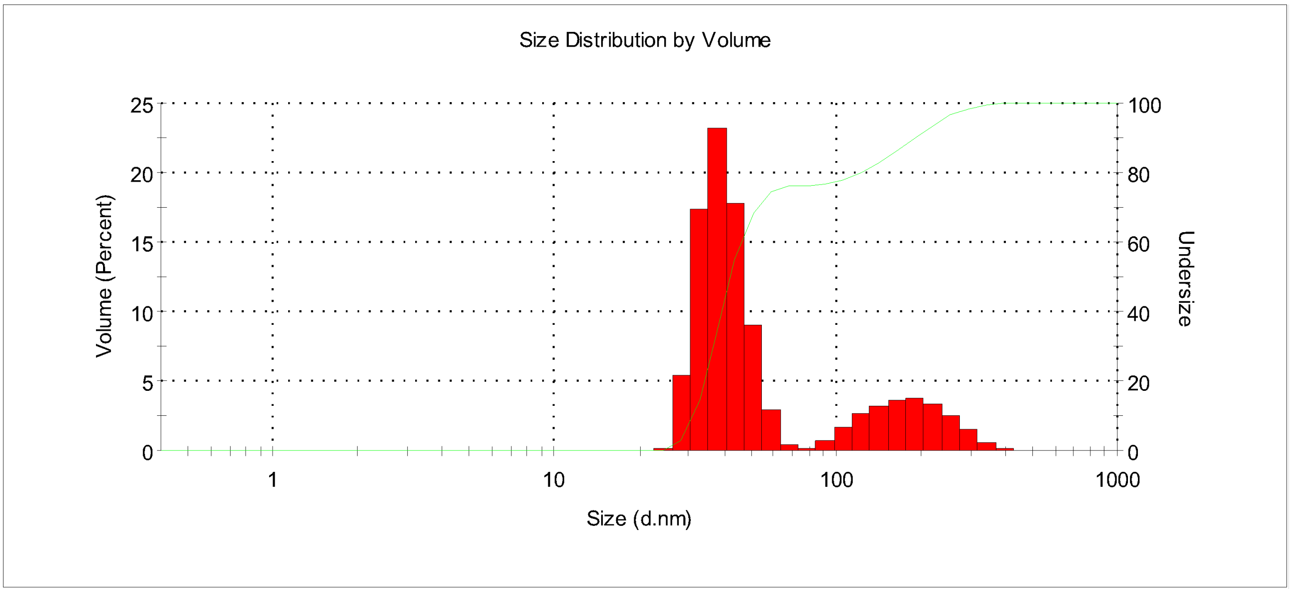

Size distribution by volume of small-EVs from ascites-derived tumor cells (PBS 1X, pH 7.4, 25°C)

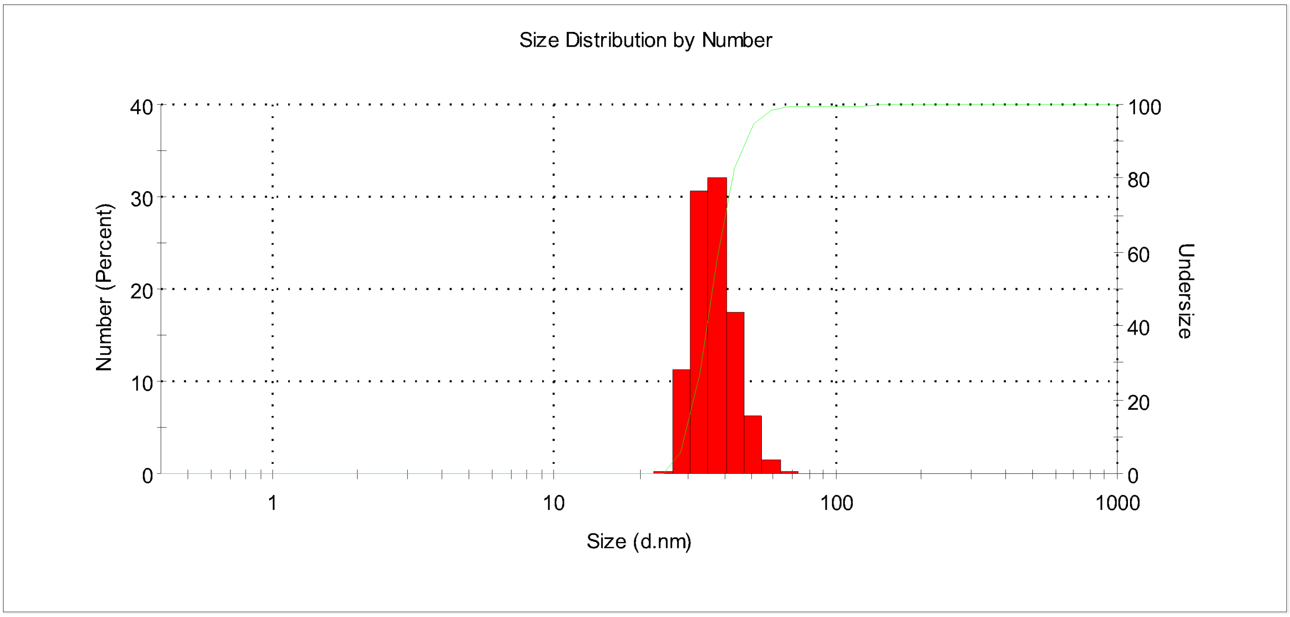

Size distribution by number of small-EVs from ascites-derived tumor cells (PBS 1X, pH 7.4, 25°C)
