# Supplementary material for: Small extracellular vesicles from malignant ascites of patients with advanced ovarian cancer provide insights into the dynamics of the extracellular matrix
Source: Mol Oncol. 2021 Oct 27;15(12):3596–614. doi: 10.1002/1878-0261.13110 (PMC8637559; doi:10.1002/1878-0261.13110)

Patient 1

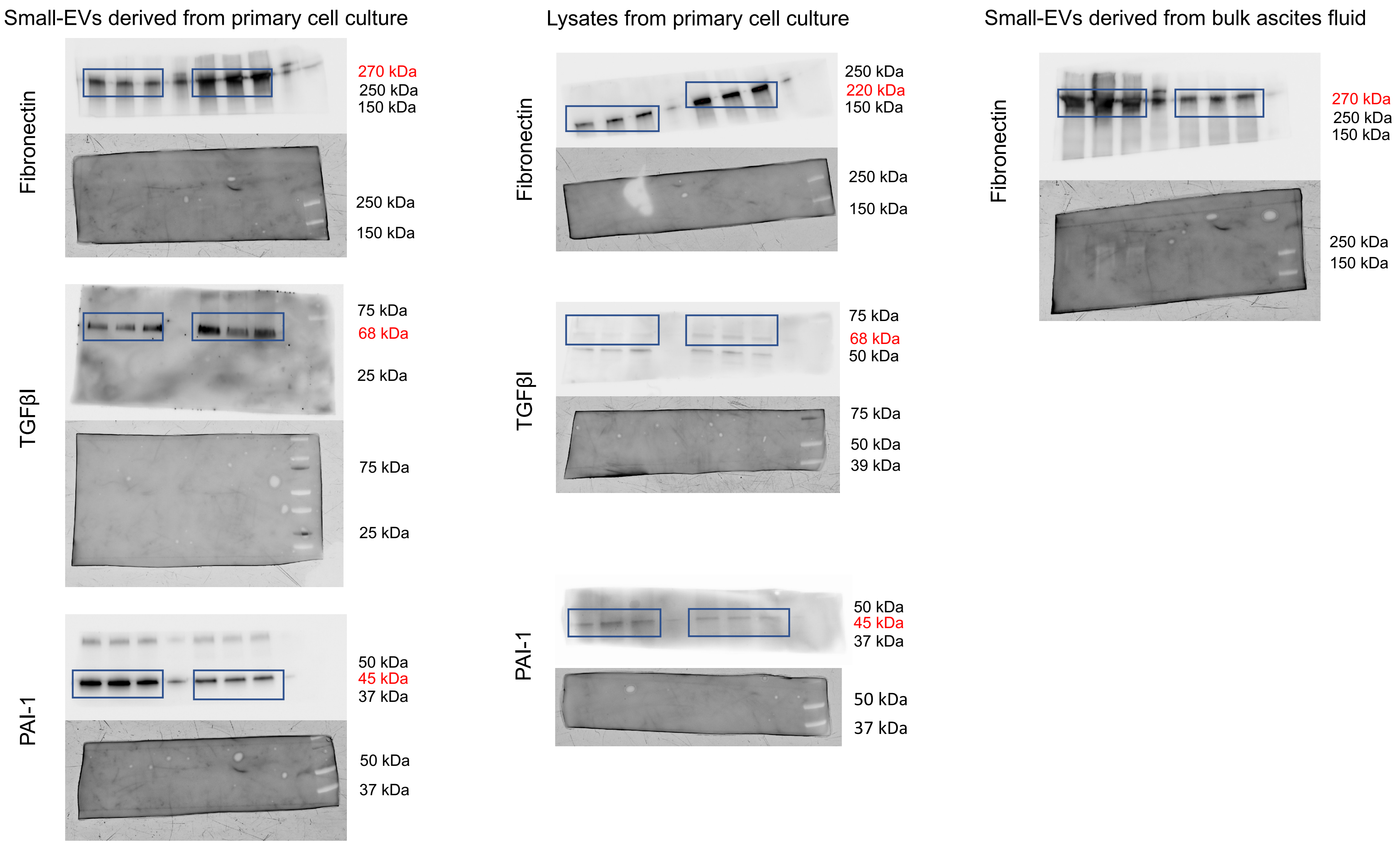
Small-EVs derived from primary cell culture
Lysates from primary cell culture
Small-EVs derived from bulk ascites fluid
Fibronectin
270 kDa
250 kDa
150 kDa
250 kDa
150 kDa
TGFβI
75 kDa
68 kDa
25 kDa
75 kDa
25 kDa
PAI-1
50 kDa
45 kDa
37 kDa
50 kDa
37 kDa
Fibronectin
250 kDa
220 kDa
150 kDa
250 kDa
150 kDa
TGFβI
75 kDa
68 kDa
50 kDa
75 kDa
50 kDa
39 kDa
PAI-1
50 kDa
45 kDa
37 kDa
50 kDa
37 kDa
Fibronectin
270 kDa
250 kDa
150 kDa
250 kDa
150 kDa

Patient 2

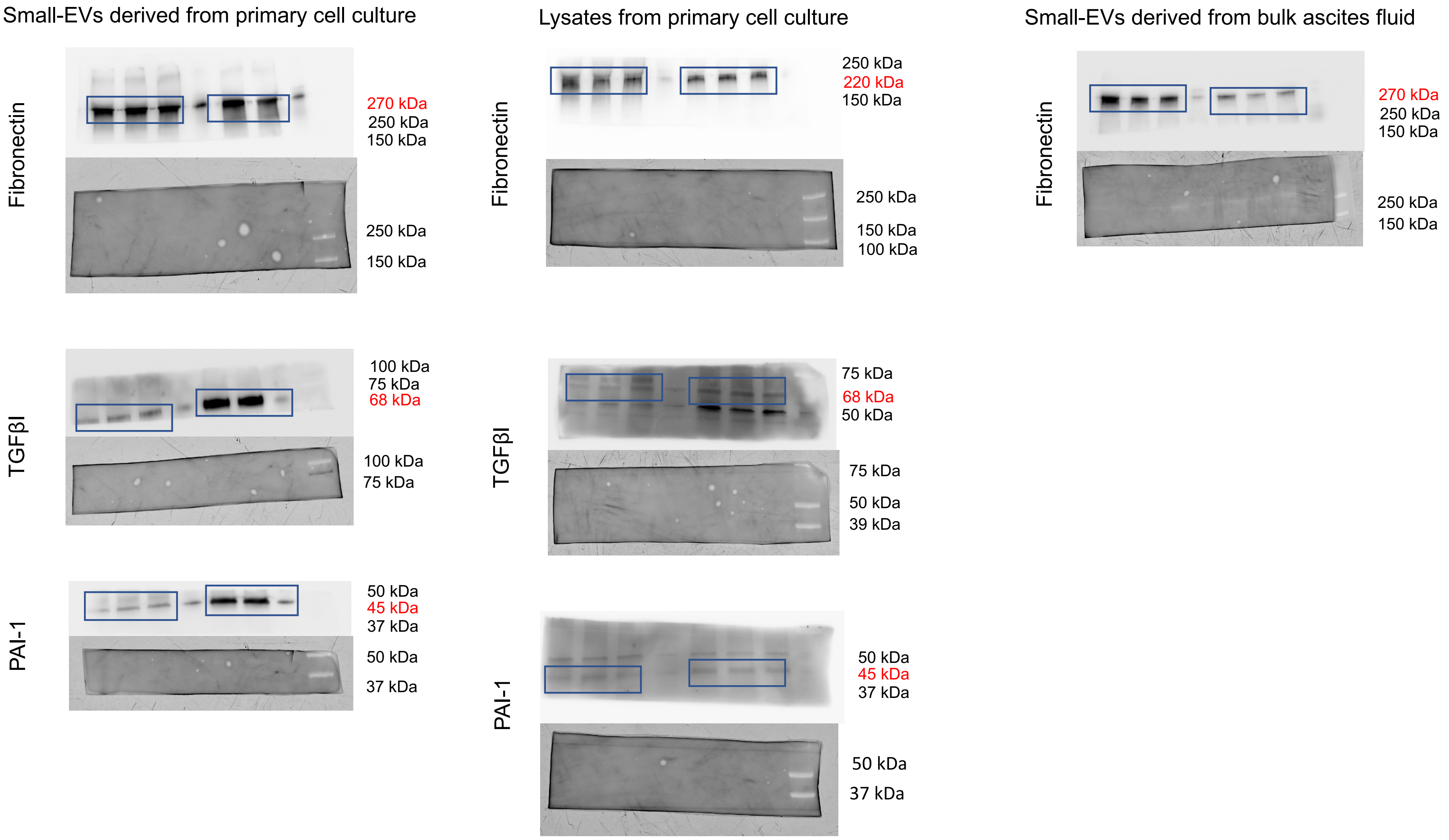

Small-EVs derived from primary cell culture
Lysates from primary cell culture
Small-EVs derived from bulk ascites fluid
Fibronectin
270 kDa
250 kDa
150 kDa
250 kDa
150 kDa
TGFβI
100 kDa
75 kDa
68 kDa
100 kDa
75 kDa
PAI-1
50 kDa
45 kDa
37 kDa
50 kDa
37 kDa
Fibronectin
250 kDa
220 kDa
150 kDa
250 kDa
150 kDa
100 kDa
TGFβI
75 kDa
68 kDa
50 kDa
75 kDa
50 kDa
39 kDa
PAI-1
50 kDa
45 kDa
37 kDa
50 kDa
37 kDa
Fibronectin
270 kDa
250 kDa
150 kDa
250 kDa
150 kDa

Patient 3

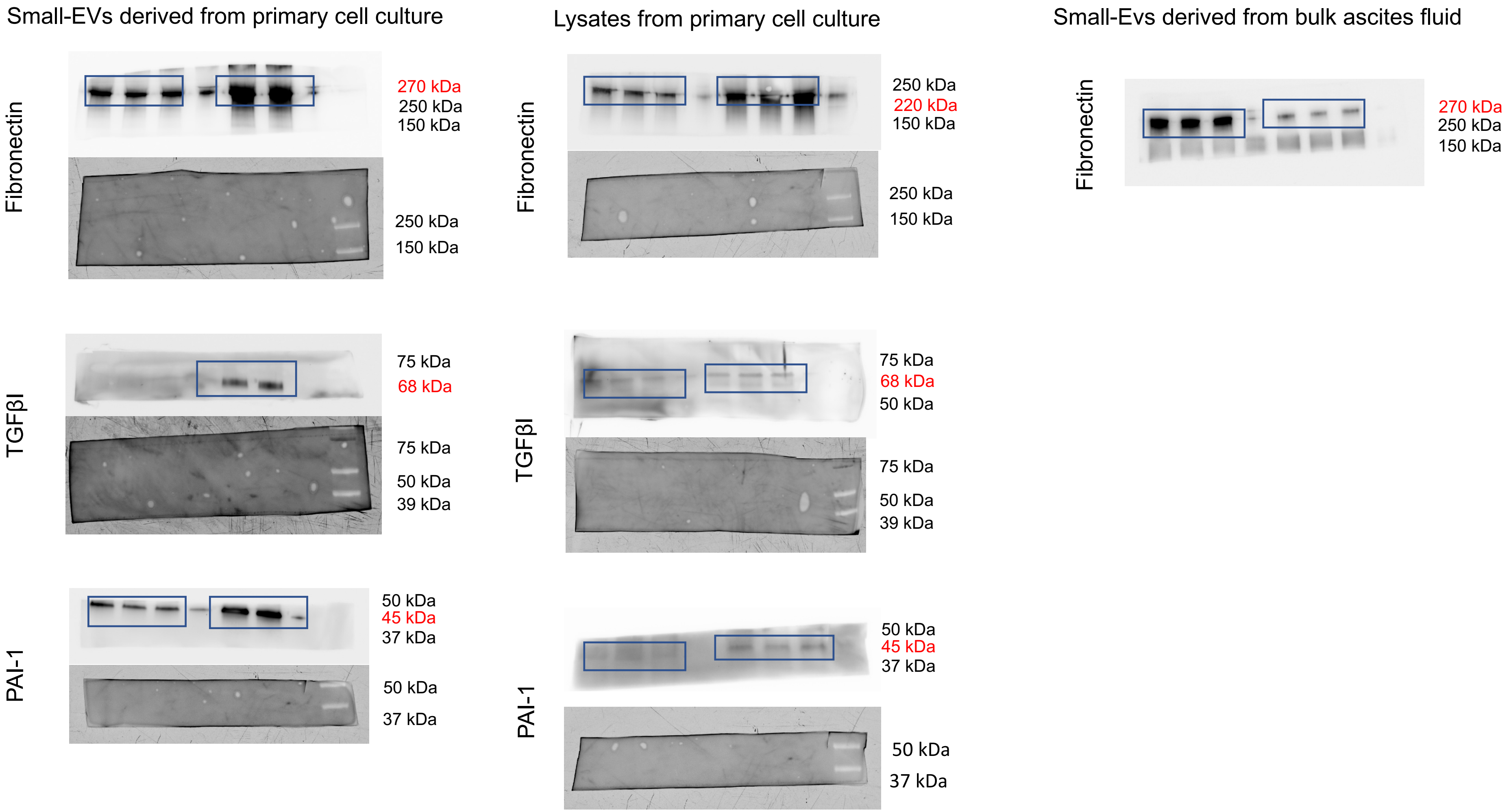

Small-EVs derived from primary cell culture
Lysates from primary cell culture
Small-Evs derived from bulk ascites fluid
Fibronectin
270 kDa
250 kDa
150 kDa
250 kDa
150 kDa
Fibronectin
250 kDa
220 kDa
150 kDa
250 kDa
150 kDa
Fibronectin
270 kDa
250 kDa
150 kDa
TGFβI
75 kDa
68 kDa
75 kDa
50 kDa
39 kDa
TGFβI
75 kDa
68 kDa
50 kDa
75 kDa
50 kDa
39 kDa
PAI-1
50 kDa
45 kDa
37 kDa
50 kDa
37 kDa
PAI-1
50 kDa
45 kDa
37 kDa
50 kDa
37 kDa

Cohort of patients before and after chemotherapy

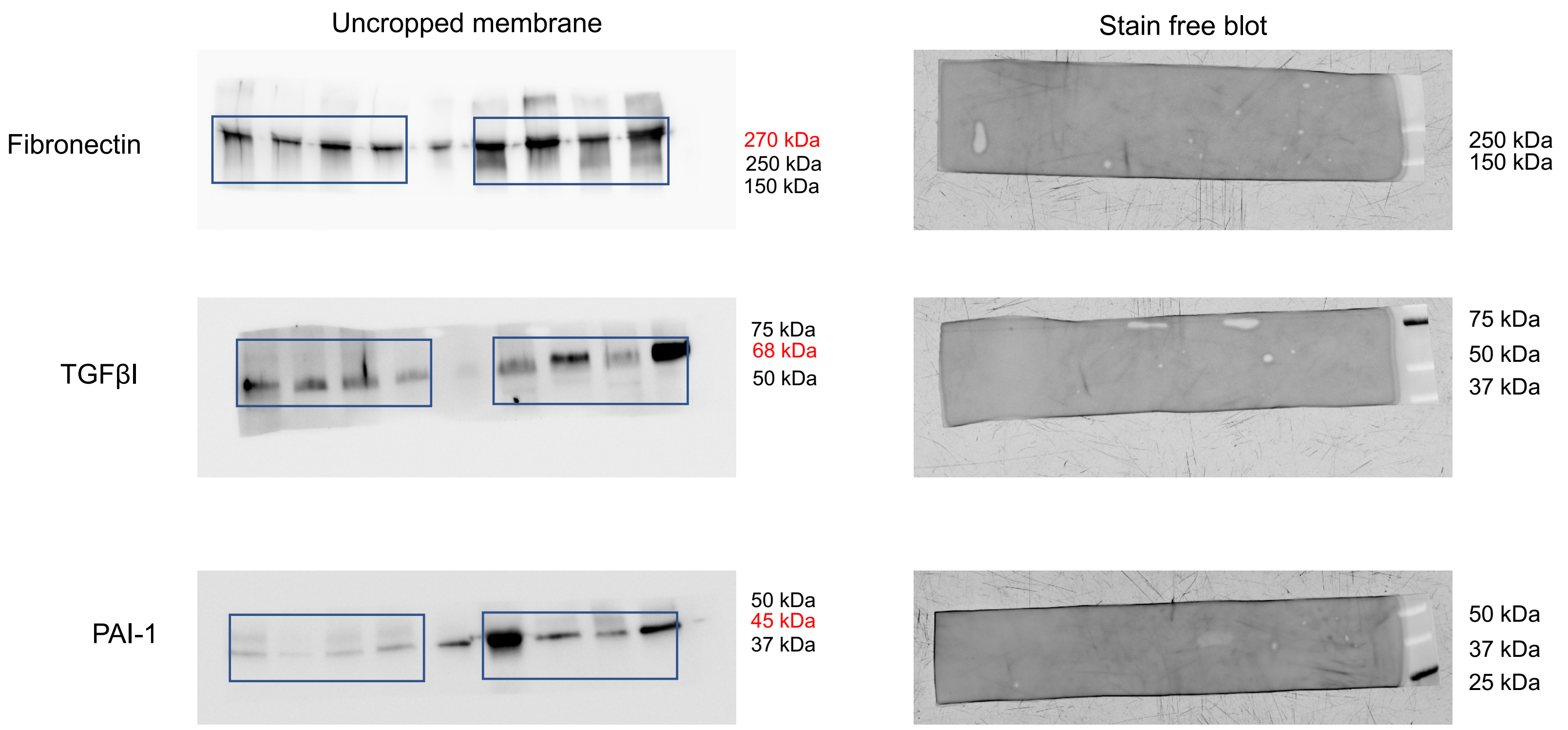

Supplement: Supplementary file 3 — Fig. S3. Uncropped full‐length pictures of Western blot membranes. [file MOL2-15-3596-s002.pdf]
