# Supplementary material for: Small extracellular vesicles from malignant ascites of patients with advanced ovarian cancer provide insights into the dynamics of the extracellular matrix
Source: Mol Oncol. 2021 Oct 27;15(12):3596–614. doi: 10.1002/1878-0261.13110 (PMC8637559; doi:10.1002/1878-0261.13110)
Supplement: Supplementary file 4 — Table S1. List of unique proteins found in small‐EVs. [file MOL2-15-3596-s001.pdf]

Bulk fluid ascites

DB	Accession	Score	Mass	Matches	Match(sig)	Sequences	Seq(sig)	emPAI	Description
SwissProt	A1AG1_HUMAN	47	23725	2	2	2	2	0.50	Alpha-1-acid glycoprotein 1 OS=Homo sapiens GN=ORM1 PE=1 SV=1
SwissProt	PON1_HUMAN	41	39877	2	2	2	2	0.28	Serum paraoxonase/arylesterase 1 OS=Homo sapiens GN=PON1 PE=1 SV=3
SwissProt	FIBA_HUMAN	38	95656	2	2	2	2	0.11	Fibrinogen alpha chain OS=Homo sapiens GN=FGA PE=1 SV=2
SwissProt	AMBP_HUMAN	84	39886	3	3	3	3	0.44	Protein AMBP OS=Homo sapiens GN=AMBP PE=1 SV=1
SwissProt	ACTB_HUMAN	105	42052	4	4	4	4	0.59	Actin, cytoplasmic 1 OS=Homo sapiens GN=ACTB PE=1 SV=1
SwissProt	KV401_HUMAN	41	13486	2	2	2	2	1.04	Immunoglobulin kappa variable 4-1 OS=Homo sapiens GN=IGKV4-1 PE=1 SV=1
SwissProt	ANGT_HUMAN	74	53406	3	3	2	2	0.20	Angiotensinogen OS=Homo sapiens GN=AGT PE=1 SV=1
SwissProt	HV333_HUMAN	33	13237	2	2	2	2	1.06	Immunoglobulin heavy variable 3-33 OS=Homo sapiens GN=IGHV3-33 PE=1 SV=2
SwissProt	KV320_HUMAN	67	12663	2	2	2	2	1.14	Immunoglobulin kappa variable 3-20 OS=Homo sapiens GN=IGKV3-20 PE=1 SV=2
SwissProt	RET4_HUMAN	50	23337	2	2	2	2	0.52	Retinol-binding protein 4 OS=Homo sapiens GN=RBP4 PE=1 SV=3
SwissProt	IGHG4_HUMAN	56	36431	4	4	4	4	0.71	Ig gamma-4 chain C region OS=Homo sapiens GN=IGHG4 PE=1 SV=1
SwissProt	CFAB_HUMAN	93	86847	7	7	7	7	0.48	Complement factor B OS=Homo sapiens GN=CFB PE=1 SV=2
SwissProt	SAMP_HUMAN	96	25485	5	5	5	5	1.58	Serum amyloid P-component OS=Homo sapiens GN=APCS PE=1 SV=2
SwissProt	FIBG_HUMAN	119	52106	4	4	4	4	0.45	Fibrinogen gamma chain OS=Homo sapiens GN=FGG PE=1 SV=3
SwissProt	ITIH1_HUMAN	70	101782	2	2	2	2	0.10	Inter-alpha-trypsin inhibitor heavy chain H1 OS=Homo sapiens GN=ITIH1 PE=1 SV=3
SwissProt	FCN3_HUMAN	59	33395	2	2	2	2	0.34	Ficolin-3 OS=Homo sapiens GN=FCN3 PE=1 SV=2
SwissProt	C4BPA_HUMAN	48	69042	3	3	3	3	0.25	C4b-binding protein alpha chain OS=Homo sapiens GN=C4BPA PE=1 SV=2
SwissProt	CO4A_HUMAN	125	194261	7	7	7	7	0.19	Complement C4-A OS=Homo sapiens GN=C4A PE=1 SV=2
SwissProt	ALBU_HUMAN	522	71317	33	33	29	29	6.38	Serum albumin OS=Homo sapiens GN=ALB PE=1 SV=2
SwissProt	PLMN_HUMAN	216	93247	9	9	9	9	0.60	Plasminogen OS=Homo sapiens GN=PLG PE=1 SV=2
SwissProt	IGHG1_HUMAN	87	36596	4	4	4	4	0.70	Ig gamma-1 chain C region OS=Homo sapiens GN=IGHG1 PE=1 SV=1
SwissProt	A2MG_HUMAN	404	164613	25	25	23	23	1.16	Alpha-2-macroglobulin OS=Homo sapiens GN=A2M PE=1 SV=3
SwissProt	IGHG2_HUMAN	119	36505	7	7	6	6	1.55	Ig gamma-2 chain C region OS=Homo sapiens GN=IGHG2 PE=1 SV=2
SwissProt	ANT3_HUMAN	64	53025	3	3	3	3	0.32	Antithrombin-III OS=Homo sapiens GN=SERPINC1 PE=1 SV=1
SwissProt	CERU_HUMAN	70	122983	4	4	4	4	0.17	Ceruloplasmin OS=Homo sapiens GN=CP PE=1 SV=1
SwissProt	HEP2_HUMAN	57	57205	3	3	3	3	0.29	Heparin cofactor 2 OS=Homo sapiens GN=SERPIND1 PE=1 SV=3
SwissProt	G3P_HUMAN	50	36201	2	2	2	2	0.31	Glyceraldehyde-3-phosphate dehydrogenase OS=Homo sapiens GN=GAPDH PE=1 SV=3
SwissProt	IC1_HUMAN	72	55347	3	3	3	3	0.30	Plasma protease C1 inhibitor OS=Homo sapiens GN=SERPING1 PE=1 SV=2
SwissProt	CRP_HUMAN	37	25194	2	2	2	2	0.47	C-reactive protein OS=Homo sapiens GN=CRP PE=1 SV=1
SwissProt	LAC2_HUMAN	50	11458	3	3	3	3	2.48	Ig lambda-2 chain C regions OS=Homo sapiens GN=IGLC2 PE=1 SV=1
SwissProt	ITIH4_HUMAN	42	103521	3	3	3	3	0.15	Inter-alpha-trypsin inhibitor heavy chain H4 OS=Homo sapiens GN=ITIH4 PE=1 SV=4
SwissProt	APOA1_HUMAN	132	30759	8	8	8	8	2.66	Apolipoprotein A-I OS=Homo sapiens GN=APOA1 PE=1 SV=1
SwissProt	HPTR_HUMAN	42	39518	2	2	2	2	0.28	Haptoglobin-related protein OS=Homo sapiens GN=HPR PE=2 SV=2
SwissProt	VTDB_HUMAN	39	54526	2	2	2	2	0.20	Vitamin D-binding protein OS=Homo sapiens GN=GC PE=1 SV=1
SwissProt	CLUS_HUMAN	30	53031	2	2	2	2	0.20	Clusterin OS=Homo sapiens GN=CLU PE=1 SV=1
SwissProt	A1AT_HUMAN	172	46878	8	8	7	7	1.30	Alpha-1-antitrypsin OS=Homo sapiens GN=SERPINA1 PE=1 SV=3
SwissProt	C1QB_HUMAN	51	26933	2	2	2	2	0.43	Complement C1q subcomponent subunit B OS=Homo sapiens GN=C1QB PE=1 SV=3
SwissProt	KVD20_HUMAN	83	12621	2	2	2	2	1.13	Immunoglobulin kappa variable 3-20 OS=Homo sapiens GN=IGKV3D-20 PE=3 SV=1
SwissProt	APOA2_HUMAN	48	11282	4	4	4	4	4.35	Apolipoprotein A-II OS=Homo sapiens GN=APOA2 PE=1 SV=1
SwissProt	IGHM_HUMAN	83	49960	5	5	5	5	0.63	Ig mu chain C region OS=Homo sapiens GN=IGHM PE=1 SV=3
SwissProt	APOC1_HUMAN	53	9326	2	2	2	2	1.76	Apolipoprotein C-I OS=Homo sapiens GN=APOC1 PE=1 SV=1
SwissProt	LBP_HUMAN	31	53521	2	2	2	2	0.20	Lipopolysaccharide-binding protein OS=Homo sapiens GN=LBP PE=1 SV=3
SwissProt	CFAI_HUMAN	45	68102	2	2	2	2	0.15	Complement factor I OS=Homo sapiens GN=CFI PE=1 SV=2
SwissProt	C1QC_HUMAN	78	25985	2	2	2	2	0.45	Complement C1q subcomponent subunit C OS=Homo sapiens GN=C1QC PE=1 SV=3
SwissProt	FHR1_HUMAN	91	38766	3	3	3	3	0.46	Complement factor H-related protein 1 OS=Homo sapiens GN=CFHR1 PE=1 SV=2
SwissProt	SAA4_HUMAN	63	14851	4	4	3	3	2.64	Serum amyloid A-4 protein OS=Homo sapiens GN=SAA4 PE=1 SV=2
SwissProt	IGKC_HUMAN	64	11773	4	4	4	4	4.03	Ig kappa chain C region OS=Homo sapiens GN=IGKC PE=1 SV=1
SwissProt	TRFE_HUMAN	169	79294	15	15	14	14	1.52	Serotransferrin OS=Homo sapiens GN=TF PE=1 SV=3
SwissProt	HEMO_HUMAN	54	52385	3	3	2	2	0.32	Hemopexin OS=Homo sapiens GN=HPX PE=1 SV=2
SwissProt	HV309_HUMAN	62	13108	2	2	2	2	1.07	Immunoglobulin heavy variable 3-9 OS=Homo sapiens GN=IGHV3-9 PE=1 SV=2
SwissProt	APOC2_HUMAN	72	11277	4	4	4	4	4.56	Apolipoprotein C-II OS=Homo sapiens GN=APOC2 PE=1 SV=1
SwissProt	HBD_HUMAN	130	16159	9	9	6	6	4.95	Hemoglobin subunit delta OS=Homo sapiens GN=HBD PE=1 SV=2
SwissProt	IGHG3_HUMAN	64	42287	2	2	2	2	0.26	Ig gamma-3 chain C region OS=Homo sapiens GN=IGHG3 PE=1 SV=2
SwissProt	IGJ_HUMAN	53	18543	2	2	2	2	0.68	Immunoglobulin J chain OS=Homo sapiens GN=JCHAIN PE=1 SV=4
SwissProt	APOD_HUMAN	45	21547	2	2	2	2	0.57	Apolipoprotein D OS=Homo sapiens GN=APOD PE=1 SV=1
SwissProt	HRG_HUMAN	98	60510	7	7	7	7	0.77	Histidine-rich glycoprotein OS=Homo sapiens GN=HRG PE=1 SV=1
SwissProt	HV307_HUMAN	38	13105	2	2	2	2	1.08	Immunoglobulin heavy variable 3-7 OS=Homo sapiens GN=IGHV3-7 PE=1 SV=2
SwissProt	LAC1_HUMAN	26	11512	2	2	2	2	1.28	Ig lambda-1 chain C regions OS=Homo sapiens GN=IGLC1 PE=1 SV=1
SwissProt	GELS_HUMAN	79	86043	5	5	5	5	0.33	Gelsolin OS=Homo sapiens GN=GSN PE=1 SV=1
SwissProt	S10A6_HUMAN	53	10230	2	2	2	2	1.52	Protein S100-A6 OS=Homo sapiens GN=S100A6 PE=1 SV=1
SwissProt	EF1A1_HUMAN	71	50451	2	2	2	2	0.21	Elongation factor 1-alpha 1 OS=Homo sapiens GN=EEF1A1 PE=1 SV=1
SwissProt	CO6_HUMAN	56	108367	3	3	3	3	0.15	Complement component C6 OS=Homo sapiens GN=C6 PE=1 SV=3
SwissProt	CO5_HUMAN	58	189897	2	2	2	2	0.05	Complement C5 OS=Homo sapiens GN=C5 PE=1 SV=4
SwissProt	APOC3_HUMAN	58	10846	3	3	2	2	1.39	Apolipoprotein C-III OS=Homo sapiens GN=APOC3 PE=1 SV=1
SwissProt	HBB_HUMAN	108	16102	7	7	6	6	4.98	Hemoglobin subunit beta OS=Homo sapiens GN=HBB PE=1 SV=2
SwissProt	IGLL5_HUMAN	79	23391	6	6	4	4	1.28	Immunoglobulin lambda-like polypeptide 5 OS=Homo sapiens GN=IGLL5 PE=2 SV=2
SwissProt	KNG1_HUMAN	59	72996	3	3	3	3	0.22	Kininogen-1 OS=Homo sapiens GN=KNG1 PE=1 SV=2
SwissProt	AACT_HUMAN	54	47792	2	2	2	2	0.23	Alpha-1-antichymotrypsin OS=Homo sapiens GN=SERPINA3 PE=1 SV=2
SwissProt	A1BG_HUMAN	55	54790	3	3	3	3	0.31	Alpha-1B-glycoprotein OS=Homo sapiens GN=A1BG PE=1 SV=4
SwissProt	ACTBM_HUMAN	66	42331	3	3	3	3	0.41	Putative beta-actin-like protein 3 OS=Homo sapiens GN=POTEKP PE=5 SV=1
SwissProt	VWF_HUMAN	78	322401	3	3	3	3	0.05	von Willebrand factor OS=Homo sapiens GN=VWF PE=1 SV=4
SwissProt	FINC_HUMAN	374	266052	21	21	20	20	0.48	Fibronectin OS=Homo sapiens GN=FN1 PE=1 SV=4
SwissProt	CO9_HUMAN	44	64615	2	2	2	2	0.16	Complement component C9 OS=Homo sapiens GN=C9 PE=1 SV=2
SwissProt	VTNC_HUMAN	42	55069	2	2	2	2	0.19	Vitronectin OS=Homo sapiens GN=VTN PE=1 SV=1
SwissProt	LDHB_HUMAN	90	36900	2	2	2	2	0.30	L-lactate dehydrogenase B chain OS=Homo sapiens GN=LDHB PE=1 SV=2
SwissProt	PLTP_HUMAN	54	54933	2	2	2	2	0.19	Phospholipid transfer protein OS=Homo sapiens GN=PLTP PE=1 SV=1
SwissProt	HBG1_HUMAN	68	16187	2	2	2	2	0.81	Hemoglobin subunit gamma-1 OS=Homo sapiens GN=HBG1 PE=1 SV=2
SwissProt	HV323_HUMAN	52	12745	2	2	2	2	1.12	Immunoglobulin heavy variable 3-23 OS=Homo sapiens GN=IGHV3-23 PE=1 SV=2
SwissProt	IGHA1_HUMAN	90	38486	4	4	4	4	0.66	Ig alpha-1 chain C region OS=Homo sapiens GN=IGHA1 PE=1 SV=2
SwissProt	APOE_HUMAN	50	36246	3	3	3	3	0.50	Apolipoprotein E OS=Homo sapiens GN=APOE PE=1 SV=1
SwissProt	KVD28_HUMAN	64	13062	2	2	2	2	1.07	Immunoglobulin kappa variable 2D-28 OS=Homo sapiens GN=IGKV2D-28 PE=1 SV=2
SwissProt	APOA4_HUMAN	55	45371	5	5	5	5	0.72	Apolipoprotein A-IV OS=Homo sapiens GN=APOA4 PE=1 SV=3
SwissProt	ACTC_HUMAN	104	42334	7	7	6	6	1.24	Actin, alpha cardiac muscle 1 OS=Homo sapiens GN=ACTC1 PE=1 SV=1
SwissProt	APOB_HUMAN	1000	516651	70	70	70	70	0.95	Apolipoprotein B-100 OS=Homo sapiens GN=APOB PE=1 SV=2
SwissProt	ITIH2_HUMAN	173	106853	9	9	8	8	0.51	Inter-alpha-trypsin inhibitor heavy chain H2 OS=Homo sapiens GN=ITIH2 PE=1 SV=2
SwissProt	CFAH_HUMAN	231	143680	10	10	10	10	0.41	Complement factor H OS=Homo sapiens GN=CFH PE=1 SV=4
SwissProt	CO7_HUMAN	93	96650	2	2	2	2	0.11	Complement component C7 OS=Homo sapiens GN=C7 PE=1 SV=2
SwissProt	TTHY_HUMAN	75	15991	3	3	3	3	1.47	Transthyretin OS=Homo sapiens GN=TTR PE=1 SV=1
SwissProt	IC1_HUMAN	86	55347	4	4	4	4	0.42	Plasma protease C1 inhibitor OS=Homo sapiens GN=SERPING1 PE=1 SV=2
SwissProt	PEDF_HUMAN	80	46454	2	2	2	2	0.23	Pigment epithelium-derived factor OS=Homo sapiens GN=SERPINF1 PE=1 SV=4
SwissProt	KLKB1_HUMAN	60	73433	2	2	2	2	0.14	Plasma kallikrein OS=Homo sapiens GN=KLKB1 PE=1 SV=1
SwissProt	HBA_HUMAN	87	15305	5	5	5	5	3.78	Hemoglobin subunit alpha OS=Homo sapiens GN=HBA1 PE=1 SV=2
SwissProt	CO3_HUMAN	111	188569	11	11	11	11	0.33	Complement C3 OS=Homo sapiens GN=C3 PE=1 SV=2
SwissProt	APOH_HUMAN	35	39584	2	2	2	2	0.28	Beta-2-glycoprotein 1 OS=Homo sapiens GN=APOH PE=1 SV=3
SwissProt	HPT_HUMAN	95	45861	5	5	5	5	0.71	Haptoglobin OS=Homo sapiens GN=HP PE=1 SV=1
SwissProt	FIBB_HUMAN	61	56577	2	2	2	2	0.19	Fibrinogen beta chain OS=Homo sapiens GN=FGB PE=1 SV=2
SwissProt	C1QA_HUMAN	57	26285	2	2	2	2	0.45	Complement C1q subcomponent subunit A OS=Homo sapiens GN=C1QA PE=1 SV=2
SwissProt	G3P_HUMAN	50	36201	2	2	2	2	0.31	Glyceraldehyde-3-phosphate dehydrogenase OS=Homo sapiens GN=GAPDH PE=1 SV=3
SwissProt	C4BPA_HUMAN	74	69042	4	4	4	4	0.33	C4b-binding protein alpha chain OS=Homo sapiens GN=C4BPA PE=1 SV=2
SwissProt	CD5L_HUMAN	67	39603	2	2	2	2	0.28	CD5 antigen-like OS=Homo sapiens GN=CD5L PE=1 SV=1
SwissProt	THRB_HUMAN	70	71475	2	2	2	2	0.15	Prothrombin OS=Homo sapiens GN=F2 PE=1 SV=2
SwissProt	LV147_HUMAN	51	12447	2	2	2	2	1.16	Immunoglobulin lambda variable 1-47 OS=Homo sapiens GN=IGLV1-47 PE=1 SV=2
SwissProt	LV208_HUMAN	47	12488	4	4	3	3	3.59	Immunoglobulin lambda variable 2-8 OS=Homo sapiens GN=IGLV2-8 PE=1 SV=2
SwissProt	FHR2_HUMAN	37	31543	3	3	2	2	0.36	Complement factor H-related protein 2 OS=Homo sapiens GN=CFHR2 PE=1 SV=1

Tumor cells cultures

DB	Accession	Score	Mass	Matches	Match(sig)	Sequences	Seq(sig)	emPAI	Description
SwissProt	NDKA_HUMAI	44	17309	2	2	2	2	0.75	Nucleoside diphosphate kinase A OS=Homo sapiens GN=NME1 PE=1 SV=1
SwissProt	TRFL_HUMAN	54	80014	2	2	2	2	0.13	Lactotransferrin OS=Homo sapiens GN=LTF PE=1 SV=6
SwissProt	ACTN4_HUMA	82	105245	3	3	3	3	0.15	Alpha-actinin-4 OS=Homo sapiens GN=ACTN4 PE=1 SV=2
SwissProt	ACTB_HUMAN	111	42052	5	5	5	5	0.79	Actin, cytoplasmic 1 OS=Homo sapiens GN=ACTB PE=1 SV=1
SwissProt	HS90B_HUMA	36	83554	2	2	2	2	0.13	Heat shock protein HSP 90-beta OS=Homo sapiens GN=HSP90AB1 PE=1 SV=4
SwissProt	ANXA2_HUMA	110	38808	8	8	7	7	1.41	Annexin A2 OS=Homo sapiens GN=ANXA2 PE=1 SV=2
SwissProt	FLNC_HUMAN	43	293407	2	2	2	2	0.03	Filamin-C OS=Homo sapiens GN=FLNC PE=1 SV=3
SwissProt	A2MG_HUMA	65	164613	4	4	4	4	0.13	Alpha-2-macroglobulin OS=Homo sapiens GN=A2M PE=1 SV=3
SwissProt	ALDOA_HUMA	118	39851	5	5	5	5	0.84	Fructose-bisphosphate aldolase A OS=Homo sapiens GN=ALDOA PE=1 SV=2
SwissProt	RL22_HUMAN	103	14835	2	2	2	2	0.91	60S ribosomal protein L22 OS=Homo sapiens GN=RPL22 PE=1 SV=2
SwissProt	ENOA_HUMAN	89	47481	6	6	6	6	0.85	Alpha-enolase OS=Homo sapiens GN=ENO1 PE=1 SV=2
SwissProt	LDHA_HUMAN	96	36950	5	5	5	5	0.94	L-lactate dehydrogenase A chain OS=Homo sapiens GN=LDHA PE=1 SV=2
SwissProt	PZP_HUMAN	62	165242	2	2	2	2	0.06	Pregnancy zone protein OS=Homo sapiens GN=PZP PE=1 SV=4
SwissProt	ITIH3_HUMAN	76	100072	3	3	3	3	0.16	Inter-alpha-trypsin inhibitor heavy chain H3 OS=Homo sapiens GN=ITIH3 PE=1 SV=2
SwissProt	PGBM_HUMA	49	479253	2	2	2	2	0.02	Basement membrane-specific heparan sulfate proteoglycan core protein OS=Homo sapiens GN=HSPG2 PE=1 SV=4
SwissProt	GDN_HUMAN	73	44202	2	2	2	2	0.25	Glia-derived nexin OS=Homo sapiens GN=SERPINE2 PE=1 SV=1
SwissProt	ENPL_HUMAN	74	92696	2	2	2	2	0.11	Endoplasmin OS=Homo sapiens GN=HSP90B1 PE=1 SV=1
SwissProt	ACTA_HUMAN	60	42381	2	2	2	2	0.26	Actin, aortic smooth muscle OS=Homo sapiens GN=ACTA2 PE=1 SV=1
SwissProt	QSOX1_HUMA	114	83324	7	7	6	6	0.51	Sulfhydryl oxidase 1 OS=Homo sapiens GN=QSOX1 PE=1 SV=3
SwissProt	NDKB_HUMAI	60	17401	5	5	5	5	2.99	Nucleoside diphosphate kinase B OS=Homo sapiens GN=NME2 PE=1 SV=1
SwissProt	LAMA5_HUMA	37	412023	2	2	2	2	0.02	Laminin subunit alpha-5 OS=Homo sapiens GN=LAMA5 PE=1 SV=8
SwissProt	GRP78_HUMA	44	72402	2	2	2	2	0.14	78 kDa glucose-regulated protein OS=Homo sapiens GN=HSPA5 PE=1 SV=2
SwissProt	CO4A_HUMAN	47	194261	2	2	2	2	0.05	Complement C4-A OS=Homo sapiens GN=C4A PE=1 SV=2
SwissProt	ALBU_HUMAN	613	71317	40	40	34	34	11.45	Serum albumin OS=Homo sapiens GN=ALB PE=1 SV=2
SwissProt	CXCL6_HUMA	40	12175	2	2	2	2	1.19	C-X-C motif chemokine 6 OS=Homo sapiens GN=CXCL6 PE=1 SV=4
SwissProt	CO1A2_HUMA	33	129749	2	2	2	2	0.08	Collagen alpha-2(I) chain OS=Homo sapiens GN=COL1A2 PE=1 SV=7
SwissProt	CYR61_HUMA	50	44165	2	2	2	2	0.25	Protein CYR61 OS=Homo sapiens GN=CYR61 PE=1 SV=1
SwissProt	AGRIN_HUMA	161	225246	5	5	5	5	0.12	Agrin OS=Homo sapiens GN=AGRN PE=1 SV=5
SwissProt	LAMA4_HUMA	44	205020	2	2	2	2	0.05	Laminin subunit alpha-4 OS=Homo sapiens GN=LAMA4 PE=1 SV=4
SwissProt	PPIB_HUMAN	58	23785	2	2	2	2	0.51	Peptidyl-prolyl cis-trans isomerase B OS=Homo sapiens GN=PPIB PE=1 SV=2
SwissProt	ANT3_HUMAN	54	53025	2	2	2	2	0.20	Antithrombin-III OS=Homo sapiens GN=SERPINC1 PE=1 SV=1
SwissProt	CO3A1_HUMA	60	139733	3	3	2	2	0.11	Collagen alpha-1(III) chain OS=Homo sapiens GN=COL3A1 PE=1 SV=4
SwissProt	ACTBL_HUMA	54	42318	3	3	3	3	0.41	Beta-actin-like protein 2 OS=Homo sapiens GN=ACTBL2 PE=1 SV=2
SwissProt	H90B2_HUMA	64	44492	2	2	2	2	0.24	Putative heat shock protein HSP 90-beta 2 OS=Homo sapiens GN=HSP90AB2P PE=1 SV=2
SwissProt	PAI1_HUMAN	61	45088	5	5	5	5	0.72	Plasminogen activator inhibitor 1 OS=Homo sapiens GN=SERPINE1 PE=1 SV=1
SwissProt	TETN_HUMAN	37	22921	2	2	2	2	0.53	Tetranectin OS=Homo sapiens GN=CLEC3B PE=1 SV=3
SwissProt	CCD80_HUMA	66	108505	2	2	2	2	0.09	Coiled-coil domain-containing protein 80 OS=Homo sapiens GN=CCDC80 PE=1 SV=1
SwissProt	LAMB1_HUMA	41	205150	2	2	2	2	0.05	Laminin subunit beta-1 OS=Homo sapiens GN=LAMB1 PE=1 SV=2
SwissProt	CO6A3_HUMA	89	345167	5	5	5	5	0.07	Collagen alpha-3(VI) chain OS=Homo sapiens GN=COL6A3 PE=1 SV=5
SwissProt	LOXL2_HUMA	47	88778	4	4	4	4	0.25	Lysyl oxidase homolog 2 OS=Homo sapiens GN=LOXL2 PE=1 SV=1
SwissProt	HSP7C_HUMA	64	71082	2	2	2	2	0.15	Heat shock cognate 71 kDa protein OS=Homo sapiens GN=HSPA8 PE=1 SV=1
SwissProt	ACTN1_HUMA	51	103563	2	2	2	2	0.10	Alpha-actinin-1 OS=Homo sapiens GN=ACTN1 PE=1 SV=2
SwissProt	CO1A1_HUMA	53	139883	2	2	2	2	0.07	Collagen alpha-1(I) chain OS=Homo sapiens GN=COL1A1 PE=1 SV=5
SwissProt	HPTR_HUMAN	83	39518	3	3	3	3	0.45	Haptoglobin-related protein OS=Homo sapiens GN=HPR PE=2 SV=2
SwissProt	VTDB_HUMAN	32	54526	2	2	2	2	0.20	Vitamin D-binding protein OS=Homo sapiens GN=GC PE=1 SV=1
SwissProt	RS19_HUMAN	56	16051	2	2	2	2	0.82	40S ribosomal protein S19 OS=Homo sapiens GN=RPS19 PE=1 SV=2
SwissProt	PTX3_HUMAN	118	42519	4	4	3	3	0.58	Pentraxin-related protein PTX3 OS=Homo sapiens GN=PTX3 PE=1 SV=3
SwissProt	POSTN_HUMA	36	93883	2	2	2	2	0.11	Periostin OS=Homo sapiens GN=POSTN PE=1 SV=2
SwissProt	LAMC1_HUMA	57	183191	2	2	2	2	0.06	Laminin subunit gamma-1 OS=Homo sapiens GN=LAMC1 PE=1 SV=3
SwissProt	ANXA1_HUMA	69	38918	2	2	2	2	0.30	Annexin A1 OS=Homo sapiens GN=ANXA1 PE=1 SV=2
SwissProt	C1R_HUMAN	53	81606	3	3	3	3	0.20	Complement C1r subcomponent OS=Homo sapiens GN=C1R PE=1 SV=2
SwissProt	PXDN_HUMAI	61	167793	2	2	2	2	0.06	Peroxidasin homolog OS=Homo sapiens GN=PXDN PE=1 SV=2
SwissProt	RAP1A_HUMA	40	21316	2	2	2	2	0.58	Ras-related protein Rap-1A OS=Homo sapiens GN=RAP1A PE=1 SV=1
SwissProt	TLN1_HUMAN	45	271766	3	3	3	3	0.06	Talin-1 OS=Homo sapiens GN=TLN1 PE=1 SV=3
SwissProt	CSTN1_HUMA	33	110978	2	2	2	2	0.09	Calsyntenin-1 OS=Homo sapiens GN=CLSTN1 PE=1 SV=1
SwissProt	EF2_HUMAN	80	96246	4	4	4	4	0.23	Elongation factor 2 OS=Homo sapiens GN=EEF2 PE=1 SV=4
SwissProt	TENX_HUMAN	27	464946	2	2	2	2	0.02	Tenascin-X OS=Homo sapiens GN=TNXB PE=1 SV=4
SwissProt	CATB_HUMAN	29	38766	2	2	2	2	0.29	Cathepsin B OS=Homo sapiens GN=CTSB PE=1 SV=3
SwissProt	G3P_HUMAN	26	36201	2	2	2	2	0.31	Glyceraldehyde-3-phosphate dehydrogenase OS=Homo sapiens GN=GAPDH PE=1 SV=3
SwissProt	CTGF_HUMAN	84	40289	2	2	2	2	0.27	Connective tissue growth factor OS=Homo sapiens GN=CTGF PE=1 SV=2
SwissProt	TBB1_HUMAN	52	50865	2	2	2	2	0.21	Tubulin beta-1 chain OS=Homo sapiens GN=TUBB1 PE=1 SV=1
SwissProt	FBLN1_HUMA	68	81268	3	3	3	3	0.20	Fibulin-1 OS=Homo sapiens GN=FBLN1 PE=1 SV=4
SwissProt	AFAM_HUMAI	63	70963	3	3	3	3	0.23	Afamin OS=Homo sapiens GN=AFM PE=1 SV=1
SwissProt	ANXA5_HUMA	65	35971	2	2	2	2	0.32	Annexin A5 OS=Homo sapiens GN=ANXA5 PE=1 SV=2
SwissProt	HEMO_HUMA	50	52385	2	2	2	2	0.20	Hemopexin OS=Homo sapiens GN=HPX PE=1 SV=2
SwissProt	TRFE_HUMAN	142	79294	8	8	8	8	0.64	Serotransferrin OS=Homo sapiens GN=TF PE=1 SV=3
SwissProt	TPA_HUMAN	97	65043	5	5	5	5	0.48	Tissue-type plasminogen activator OS=Homo sapiens GN=PLAT PE=1 SV=1
SwissProt	SAHH_HUMAN	51	48255	2	2	2	2	0.22	Adenosylhomocysteinase OS=Homo sapiens GN=AHCY PE=1 SV=4
SwissProt	GELS_HUMAN	138	86043	7	7	7	7	0.49	Gelsolin OS=Homo sapiens GN=GSN PE=1 SV=1
SwissProt	EF1A1_HUMA	65	50451	2	2	2	2	0.21	Elongation factor 1-alpha 1 OS=Homo sapiens GN=EEF1A1 PE=1 SV=1
SwissProt	CO5_HUMAN	47	189897	2	2	2	2	0.05	Complement C5 OS=Homo sapiens GN=C5 PE=1 SV=4
SwissProt	MRC2_HUMA	40	169761	2	2	2	2	0.06	C-type mannose receptor 2 OS=Homo sapiens GN=MRC2 PE=1 SV=2
SwissProt	HBB_HUMAN	35	16102	2	2	2	2	0.82	Hemoglobin subunit beta OS=Homo sapiens GN=HBB PE=1 SV=2
SwissProt	A1BG_HUMAN	45	54790	3	3	3	3	0.31	Alpha-1B-glycoprotein OS=Homo sapiens GN=A1BG PE=1 SV=4
SwissProt	COMP_HUMA	63	85431	2	2	2	2	0.12	Cartilage oligomeric matrix protein OS=Homo sapiens GN=COMP PE=1 SV=2
SwissProt	ACTBM_HUMA	72	42331	3	3	3	3	0.41	Putative beta-actin-like protein 3 OS=Homo sapiens GN=POTEKP PE=5 SV=1
SwissProt	TFPI2_HUMAN	42	27942	2	2	2	2	0.41	Tissue factor pathway inhibitor 2 OS=Homo sapiens GN=TFPI2 PE=1 SV=1
SwissProt	FINC_HUMAN	427	266052	23	23	22	22	0.53	Fibronectin OS=Homo sapiens GN=FN1 PE=1 SV=4
SwissProt	TSP1_HUMAN	89	133291	4	4	4	4	0.16	Thrombospondin-1 OS=Homo sapiens GN=THBS1 PE=1 SV=2
SwissProt	LDHB_HUMAN	70	36900	2	2	2	2	0.30	L-lactate dehydrogenase B chain OS=Homo sapiens GN=LDHB PE=1 SV=2
SwissProt	S10A6_HUMA	56	10230	2	2	2	2	1.53	Protein S100-A6 OS=Homo sapiens GN=S100A6 PE=1 SV=1
SwissProt	GBB1_HUMAN	59	38151	2	2	2	2	0.29	Guanine nucleotide-binding protein G(I)/G(S)/G(T) subunit beta-1 OS=Homo sapiens GN=GNB1 PE=1 SV=3
SwissProt	ITB1_HUMAN	51	91664	2	2	2	2	0.11	Integrin beta-1 OS=Homo sapiens GN=ITGB1 PE=1 SV=2
SwissProt	HS90A_HUMA	71	85006	3	3	3	3	0.19	Heat shock protein HSP 90-alpha OS=Homo sapiens GN=HSP90AA1 PE=1 SV=5
SwissProt	MOES_HUMAI	71	67892	4	4	4	4	0.34	Moesin OS=Homo sapiens GN=MSN PE=1 SV=3
SwissProt	FBLN3_HUMA	77	56885	2	2	2	2	0.19	EGF-containing fibulin-like extracellular matrix protein 1 OS=Homo sapiens GN=EFEMP1 PE=1 SV=2
SwissProt	FLNB_HUMAN	39	280157	2	2	2	2	0.04	Filamin-B OS=Homo sapiens GN=FLNB PE=1 SV=2
SwissProt	PRDX2_HUMA	41	22049	2	2	2	2	0.57	Peroxiredoxin-2 OS=Homo sapiens GN=PRDX2 PE=1 SV=5
SwissProt	FBN1_HUMAN	71	332664	2	2	2	2	0.03	Fibrillin-1 OS=Homo sapiens GN=FBN1 PE=1 SV=3
SwissProt	CO6A1_HUMA	61	109602	3	3	3	3	0.14	Collagen alpha-1(VI) chain OS=Homo sapiens GN=COL6A1 PE=1 SV=3
SwissProt	VIME_HUMAN	54	53676	2	2	2	2	0.20	Vimentin OS=Homo sapiens GN=VIM PE=1 SV=4
SwissProt	BGH3_HUMAI	135	75261	8	8	8	8	0.69	Transforming growth factor-beta-induced protein ig-h3 OS=Homo sapiens GN=TGFBI PE=1 SV=1
SwissProt	TIMP1_HUMA	53	23840	2	2	2	2	0.50	Metalloproteinase inhibitor 1 OS=Homo sapiens GN=TIMP1 PE=1 SV=1
SwissProt	APOB_HUMAN	57	516651	2	2	2	2	0.02	Apolipoprotein B-100 OS=Homo sapiens GN=APOB PE=1 SV=2
SwissProt	ITIH2_HUMAN	46	106853	2	2	2	2	0.10	Inter-alpha-trypsin inhibitor heavy chain H2 OS=Homo sapiens GN=ITIH2 PE=1 SV=2
SwissProt	H2A1B_HUMA	45	14127	2	2	2	2	0.99	Histone H2A type 1-B/E OS=Homo sapiens GN=HIST1H2AB PE=1 SV=2
SwissProt	PEDF_HUMAN	52	46454	2	2	2	2	0.23	Pigment epithelium-derived factor OS=Homo sapiens GN=SERPINF1 PE=1 SV=4
SwissProt	LG3BP_HUMA	124	66202	5	5	5	5	0.45	Galectin-3-binding protein OS=Homo sapiens GN=LGALS3BP PE=1 SV=1
SwissProt	CO3_HUMAN	327	188569	18	18	18	18	0.60	Complement C3 OS=Homo sapiens GN=C3 PE=1 SV=2
SwissProt	HBA_HUMAN	74	15305	4	4	4	4	2.49	Hemoglobin subunit alpha OS=Homo sapiens GN=HBA1 PE=1 SV=2
SwissProt	PRDX1_HUMA	48	22324	2	2	2	2	0.54	Peroxiredoxin-1 OS=Homo sapiens GN=PRDX1 PE=1 SV=1
SwissProt	POTEE_HUMA	72	122882	3	3	3	3	0.13	POTE ankyrin domain family member E OS=Homo sapiens GN=POTEE PE=2 SV=3
SwissProt	APOH_HUMAI	75	39584	2	2	2	2	0.28	Beta-2-glycoprotein 1 OS=Homo sapiens GN=APOH PE=1 SV=3
SwissProt	HPT_HUMAN	62	45861	3	3	3	3	0.38	Haptoglobin OS=Homo sapiens GN=HP PE=1 SV=1
SwissProt	TSP2_HUMAN	62	133785	3	3	3	3	0.12	Thrombospondin-2 OS=Homo sapiens GN=THBS2 PE=1 SV=2
SwissProt	EZRI_HUMAN	40	69484	2	2	2	2	0.15	Ezrin OS=Homo sapiens GN=EZR PE=1 SV=4
SwissProt	PLEC_HUMAN	37	533462	2	2	2	2	0.02	Plectin OS=Homo sapiens GN=PLEC PE=1 SV=3
SwissProt	FGF2_HUMAN	36	31093	2	2	2	2	0.37	Fibroblast growth factor 2 OS=Homo sapiens GN=FGF2 PE=1 SV=3
SwissProt	FETUA_HUMA	38	40098	2	2	2	2	0.29	Alpha-2-HS-glycoprotein OS=Homo sapiens GN=AHSG PE=1 SV=1
